# Distance Matters: Biasing Mechanism, Transfer of Asymmetry, and Stereomutation in N-Annulated Perylene Bisimide Supramolecular Polymers

**DOI:** 10.1021/jacs.1c06125

**Published:** 2021-08-11

**Authors:** Manuel A. Martínez, Azahara Doncel-Giménez, Jesús Cerdá, Joaquín Calbo, Rafael Rodríguez, Juan Aragó, Jeanne Crassous, Enrique Ortí, Luis Sánchez

**Affiliations:** † Departamento de Química Orgánica, Facultad de Ciencias Químicas, Universidad Complutense de Madrid, 28040 Madrid, Spain; ‡ Instituto de Ciencia Molecular (ICMol), Universidad de Valencia, c/Catedrático José Beltrán, 2, 46980 Paterna, Spain; § Univ Rennes, CNRS, ISCR (Institut des Sciences Chimiques de Rennes), UMR 6226, F-35000 Rennes, France

## Abstract

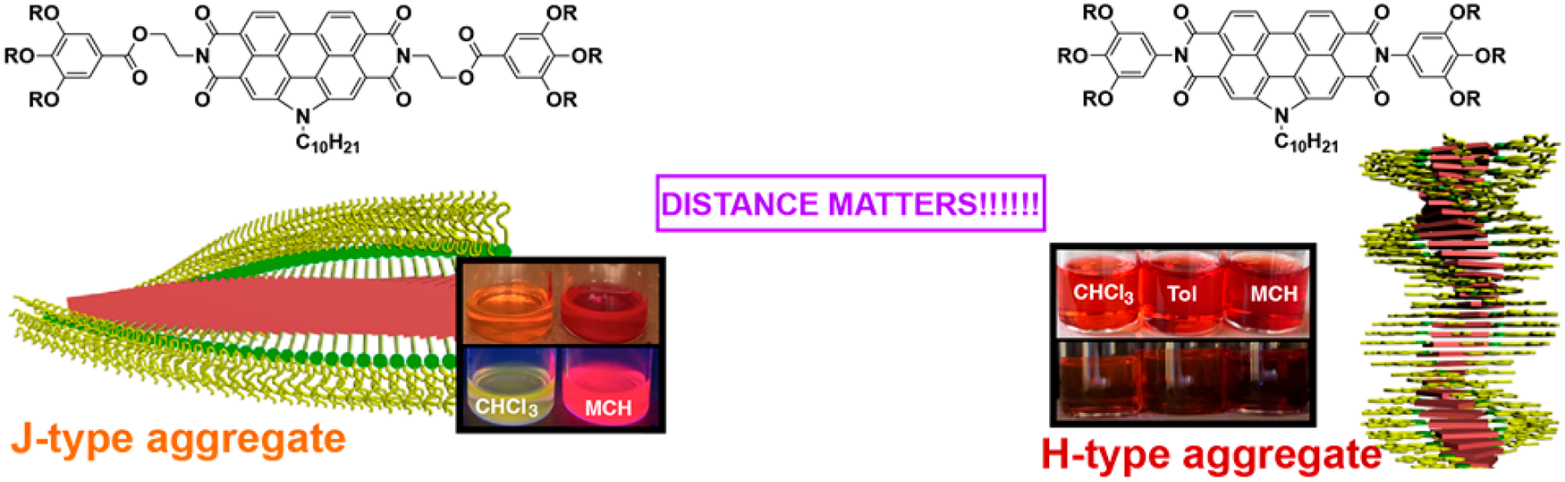

The synthesis of two series of N-annulated perylene bisimides (PBIs), compounds **1** and **2**, is reported, and their self-assembling features are thoroughly investigated by a complete set of spectroscopic measurements and theoretical calculations. The study corroborates the enormous influence that the distance between the PBI core and the peripheral groups exerts on the chiroptical properties and the supramolecular polymerization mechanism. Compounds **1**, with the peripheral groups separated from the central PBI core by two methylenes and an ester group, form J-type supramolecular polymers in a cooperative manner but exhibit negligible chiroptical properties. The lack of clear helicity, due to the staircase arrangement of the self-assembling units in the aggregate, justifies these features. In contrast, attaching the peripheral groups directly to the N-annulated PBI core drastically changes the self-assembling properties of compounds **2**, which form H-type aggregates following an isodesmic mechanism. These H-type aggregates show a strong aggregation-caused quenching (ACQ) effect that leads to nonemissive aggregates. Chiral **(*S*)-2** and **(*R*)-2** experience an efficient transfer of asymmetry to afford P*-* and M-type aggregates, respectively, although no amplification of asymmetry is achieved in majority rules or “sergeants-and-soldiers” experiments. A solvent-controlled stereomutation is observed for chiral **(*S*)-2** and **(*R*)-2**, which form helical supramolecular polymers of different handedness depending on the solvent (methylcyclohexane or toluene). The stereomutation is accounted for by considering the two possible conformations of the terminal phenyl groups, eclipsed or staggered, which lead to linear or helical self-assemblies, respectively, with different relative stabilities depending on the solvent.

## Introduction

The molecular recognition by complementary triple hydrogen bonding between diacylaminopyridines and uracil derivatives reported by Lehn and co-workers paved the way to boost the discipline of supramolecular polymerization (SP).^1^ This seminal work, supported by the discovery of J-type aggregates made by Scheibe and Jelley^2^ and the development by Klug and co-workers of the transmission electron microscopy,^3^ prompted an extraordinary progress in the field of supramolecular polymers.^4^ A key issue in the further development of functional supramolecular polymers was the establishment of mathematical models allowing for an accurate description of the SP process.^5^ Two main mechanisms have been amply described to govern the supramolecular polymerization of discrete self-assembling units. The first one, termed *isodesmic* and comparable to covalent step polymerization, is characterized by a single binding equilibrium constant for all of the supramolecular reactions yielding the final supramolecular polymer.^5^ The second one, named *cooperative* and related to covalent chain polymerization, features two binding constants for the extreme regimes of *nucleation* (*K*_n_) and *elongation* (*K*_e_).^5^ In the nucleation regime, very short aggregates are formed, which, upon overcoming a critical concentration or temperature, elongate to yield the final supramolecular polymer. However, in a cooperative mechanism with *K*_e_ ≫ *K*_n_, the opposite situation is also possible, producing an *anticooperative* mechanism.^5^ It is worth noting that the investigation of the SP mechanism requires a thermodynamic control of the process. In this regard, a number of exciting examples of pathway complexity, in which a single self-assembling unit is able to generate different aggregates in a competitive or consecutive manner, have been reported in the literature during the past few years.^6^

An intriguing question concerning the investigation of supramolecular polymers relies on establishing clear structure/function relationships that allow predicting and controlling the mechanistic pathway. The first examples of cooperative supramolecular polymers involved (i) the formation of intermolecular H-bonding arrays, (ii) the subsequent conformational changes experienced by the self-assembling units to form these H-bonding interactions, and (iii) the synergy of different noncovalent forces (H-bonding, π-stacking, hydrophobic interactions, etc.).^7^ These structural requirements were adopted as general rules to attain cooperative pathways. Importantly, George and co-workers demonstrated that permanent dipole–dipole interactions in the direction of polymer growth are the driving force for cooperative mechanisms.^8^ In this sense, some examples of cooperative supramolecular polymers that do not involve H-bonding interactions have been reported.^9^

Supramolecular polymers have found significant applications in different topics. One of these applications relies on the efficient achievement of supramolecular chirality and, more specifically, the generation of helical supramolecular structures.^10^ Supramolecular chirality is usually obtained by the efficient transfer of asymmetry from the molecular to the supramolecular level, starting from self-assembling units bearing elements of asymmetry (point or/and axial chirality).^11^ Amplification of asymmetry, by using sergeants-and-soldiers (SaS) or majority rules (MR) experiments, is an efficient strategy to attain helical homopolymers from the coassembly of different entities.^12^ Importantly, a cooperative mechanism is behind most of the examples of transfer and amplification of asymmetry in supramolecular polymers, with very few examples being reported for helical supramolecular polymers formed by following isodesmic or weakly cooperative processes.^13^

**Figure 1:**
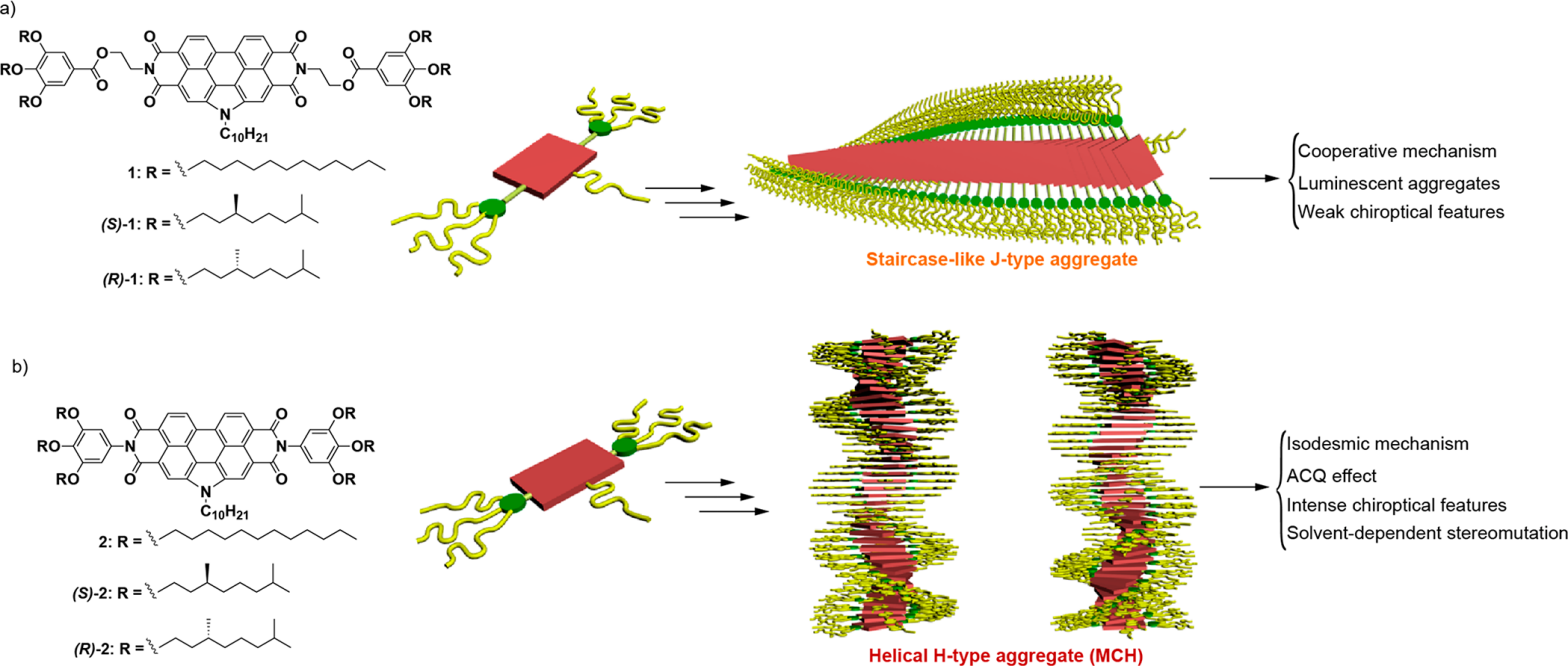
Chemical structure of the N-annulated PBIs **1** (a) and **2** (b) displaying the formation of staircase-like and helical aggregates, respectively, and the derived features of the resulting supramolecular polymers.

Herein, we report on the relevant role exerted by the chemical nature and the flexibility of the bridging units separating the peripheral side chains and the central aromatic core constituting the N-annulated perylene bisimide (PBIs) self-assembling units in the supramolecular polymerization (SP) of compounds **1** and **2** (Figure 1). While compounds **1** readily form highly luminescent, charge transfer (CT) mediated J-type aggregates in a cooperative manner,^14^ compounds **2** self-assemble into H-type aggregates following an isodesmic mechanism that results in a strong aggregation-caused quenching (ACQ).^15^ The J-type aggregates formed from chiral **(*S*)-1** and **(*R*)-1** exhibit negligible chiroptical properties, as demonstrated by electronic (ECD) and vibrational (VCD) circular dichroism studies, and a lack of circularly polarized luminescence (CPL). In contrast, the intense and bisignated ECD and VCD spectra displayed by the H-type aggregates formed by **(*S*)-2** and **(*R*)-2** reveal the formation of helical aggregates with no CPL emission due to the ACQ effect. In addition, chiral **(*S*)-2** and **(*R*)-2** present a solvent-dependent stereomutation, which can be rationalized by considering the formation of two different self-assemblies (linear vs helical) with opposite stabilities depending on the solvent conditions. The synergy of experimental evidence and theoretical calculations has been used to further justify these findings. Thus, for compounds **1**, the complementarity between the π-stacking of the aromatic PBI cores and the electrostatic forces between the peripheral benzoate groups favors the formation of staircase-like aggregates with a nonhelical long-axis-displaced supramolecular structure, leading to negligible chiroptical properties for the resulting J-type aggregates. In contrast, the parallel arrangement of the PBI units, conditioned by the eclipsed or staggered conformation of the peripheral trialkoxyphenyl moieties directly attached to the PBI core in **2**, is responsible for the formation of H-type aggregates that experience a solvent-dependent stereomutation. The studies presented in this manuscript contribute to the elaboration of structure/function relationships that are useful to predict relevant features of supramolecular polymers.

## Results and Discussion

### Synthesis and Supramolecular Polymerization Mechanism

The synthesis of N-annulated PBIs **1** and **2** was accomplished by following a procedure similar to that reported for achiral **1**, in which the corresponding chiral 2-aminoethyl 3,4,5-trialkoxybenzoate **9** (for **(*S*)-1** and **(*R*)-1**) or 3,4,5-trialkoxyaniline **14** (for **2**, **(*S*)-2**, and **(*R*)-2**) was reacted with N-annulated perylene-3,4,9,10-tetracarboxylic dianhydride **5** in the presence of Zn(AcO)_2_ and imidazole.^14^ All of the new compounds were characterized by common spectroscopic techniques. A complete description of the synthetic protocols and the spectroscopic characterization is included in the Supporting Information.

**Figure 2:**
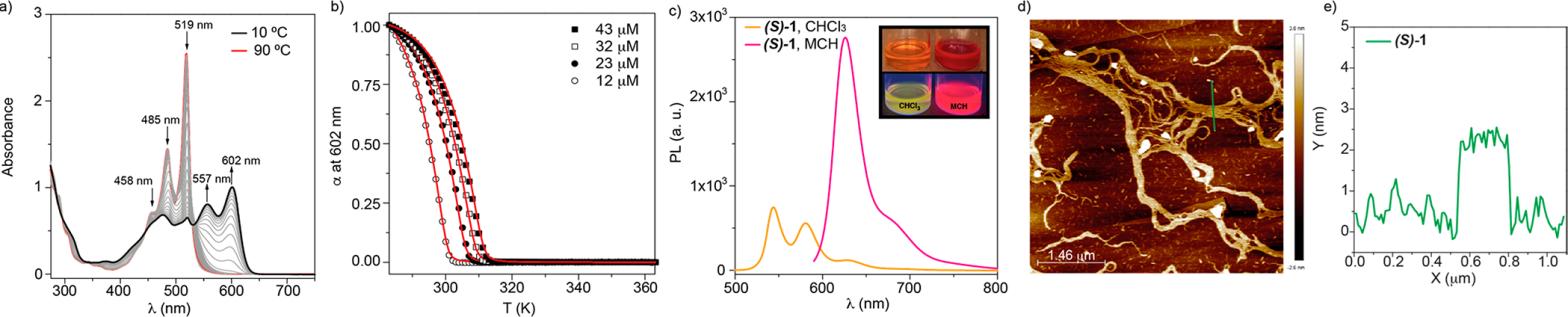
(a) UV–vis spectra of **(*S*)-1** at different temperatures (MCH, *c*_T_ = 32 μM). Arrows indicate the absorption changes upon decreasing the temperature from 90 to 10 °C. (b) Plot of the variation of the degree of polymerization (α) of **(*S*)-1** in MCH versus temperature on cooling at 1 °C min^–1^. Red curves correspond to the fitting to the EQ model. (c) Photoluminescence (PL) spectra of **(*S*)-1** in MCH (λ_exc_ = 555 nm) and CHCl_3_ (λ_exc_ = 495 nm) (*c*_T_ = 10 μM, 298 K). The inset shows the pictures of the solutions of **(*S*)-1** in MCH and CHCl_3_ used to register the PL spectra without (top samples) and with UV lamp illumination (bottom samples). (d) AFM image and (e) height profile along the green line in (d) of the bundles of fibrillar supramolecular polymers formed by **(*S*)-1** in MCH onto HOPG (spin coating, *c*_T_ = 10 μM, 298 K).

A detailed investigation of the supramolecular polymerization mechanism of the chiral **(*S*)-1** and **(*R*)-1** was performed, resting on the previous results on achiral **1**.^14^ The synergy of experimental and theoretical studies on **1** demonstrated the formation of J-type aggregates by the π-stacking of the aromatic moieties with the pyrrolic rings of the adjacent monomers pointing to the same direction. These noncovalent forces are also operative in the self-assembly of chiral **(*S*)-1** and **(*R*)-1** (Figure S1). Variable-temperature (VT) UV–vis experiments in methylcyclohexane (MCH) as solvent proved that the supramolecular polymerization mechanism of **1** occurs in a cooperative manner under thermodynamic control, since no thermal hysteresis is observed in the cooling and heating curves.^14^ In good analogy, chiral **(*S*)-1** and **(*R*)-1** present similar self-assembling features (Figure S2a). Thus, the monomeric state of these chiral N-annulated PBIs, attained by heating up diluted solutions of these compounds at 90 °C, shows two absorption peaks at 485 and 519 nm, ascribable to the A_0–1_/A_0–0_ transitions (Figure 2a).^16^ This absorption pattern coincides with that observed by using a “good” solvent such as chloroform (Figure S2b). Cooling these solutions provokes the red shift of the absorption bands to 557 and 602 nm, diagnostic of the formation of J-type aggregates (Figure 2a). The depletion of the absorption peaks at 485 and 519 nm and the increase in the peaks at 557 and 602 nm upon cooling presents a nonsigmoidal shape characteristic of a cooperative mechanism. The global fitting of the variation of the degree of aggregation α versus temperature to the equilibrium (EQ) model^17^ (Figure 2b and Figure S3) allows derivation of all the thermodynamic parameters (nucleation (Δ*H*_n_) and elongation (Δ*H*_e_) enthalpies, entropy (Δ*S*), nucleation (*K*_n_) and elongation (*K*_e_) binding constants, and the degree of cooperativity σ) associated with the supramolecular polymerization of these chiral compounds, which are similar to those derived for achiral **1** (Table 1).^14^

**Table 1:** Thermodynamic Parameters of **(*S*)-1**, **(*R*)-1**, **2**, and **(*S*)-2**

	**(*S*)-1**^a^	**(*R*)-1**^a^	**2**^b^	**(*S*)-2**^b^
Δ*H*_e_ (kJ mol^–1^)	–91.4 ± 0.5	–93.9 ± 0.4	–77.0 ± 0.7	–78.9 ± 0.6
Δ*S* (J mol^–1^)	–211 ± 1	–219 ± 1	–137 ± 2	–141 ± 1
Δ*H*_n_ (kJ mol^–1^)^c^	–16.2 ± 0.2	–16.5 ± 0.2	–77.0 ± 0.7^d^	–78.9 ± 0.6^d^
σ^c^	1.2 × 10^–3^	1.1 × 10^–3^	1	1
*K*_e_ (L mol^–1^)^c^	1.9 × 10^5^	2.0 × 10^5^	3.7 × 10^6^	5.1 × 10^6^
*K*_n_ (L mol^–1^)^c^	2.4 × 10^2^	2.2 × 10^2^		

a In MCH.

b In decalin.

c The equilibrium constants for elongation (*K*_e_) and dimerization (*K*_n_) and the cooperativity factor σ (=*K*_n_/*K*_e_) were calculated at 298 K.

d When the isodesmic nature of the supramolecular polymerization of compounds **2** is considered, the values of Δ*H*_e_ and Δ*H*_n_ are equal.

The formation of J-type aggregates for compounds **1** has also been confirmed by the intense emission of a MCH solution of **(*S*)-1** at *c*_T_ = 10 μM (Figure 2c). The fluorescence spectrum shows a sharp band centered at 626 nm with a shoulder at 684 nm, which presents a small Stokes shift between the absorption and the emission maxima of 24 nm, characteristic of J-type aggregates.^18^ The photoluminescent quantum yield (Φ_lum_) and the lifetime (τ) estimated for the aggregated species show values of 0.716 and 5.7 ns, respectively (Table S1). These emission features contrast with those observed in CHCl_3_, in which a much less intense spectrum, displaying maxima at 543 and 581 nm, is observed with lower values of Φ_lum_ (0.191) and τ (1.91 ns) (Figure 2c and Table S1). The aggregation of these N-annulated perylenes is recognizable by the naked eye through a color change of the solution from orange to deep red, with a clear change in the fluorescence under illumination (Figure 2c, inset). The morphology of the aggregates formed upon the supramolecular polymerization of **(*S*)-1** was visualized by atomic force microscopy (AFM) imaging employing highly oriented pyrolytic graphite (HOPG) as the surface. The AFM images recorded for the samples deposited onto HOPG show the formation of fibrillar aggregates bundled in highly uniform strips constituted by fibers with a typical height of 2.5 nm (Figure 2d,e and Figure S4).

**Figure 3:**
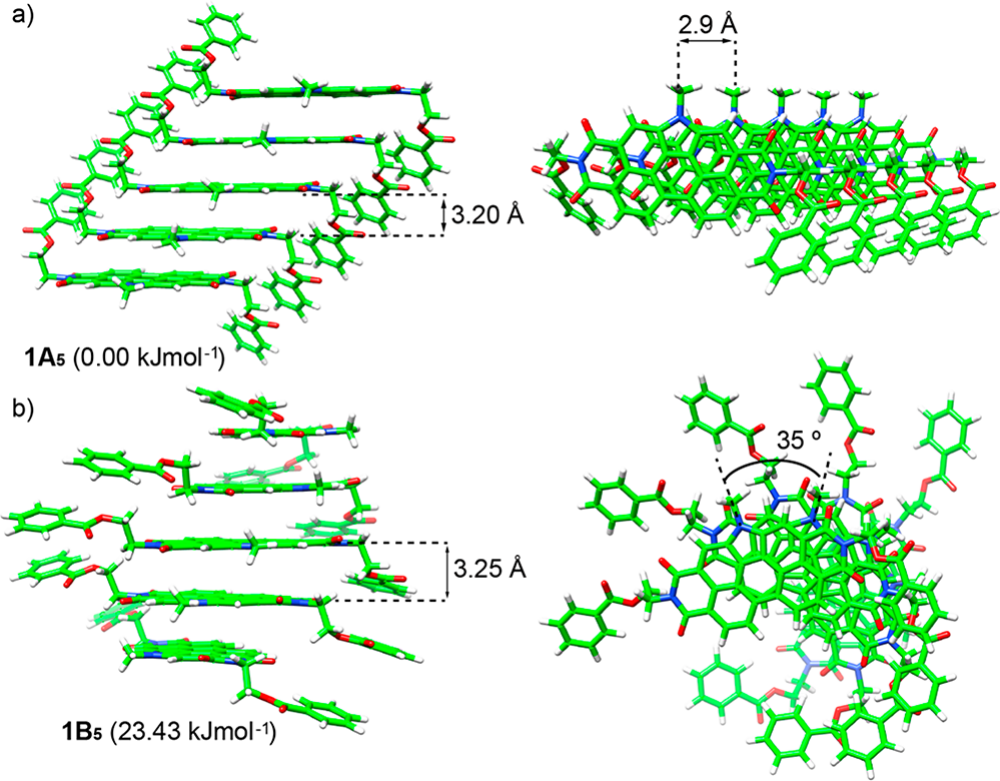
Minimum-energy structures (with their relative energies indicated) calculated at the GFN2-xTB level for the most stable pentamers **1A**_**5**_ (a) and **1B**_**5**_ (b) in the self-assembly of **1**.

To investigate the geometrical structure of the J-type aggregates, a conformational theoretical study on a simplified monomer of **1**, bearing flexible ethyl benzoate peripheral groups and methyl groups on the amine nitrogens, was first performed at the semiempirical GFN2-xTB level using the conformer–rotamer ensemble sampling tool (CREST).^19^ Among all the structural possibilities and despite the higher stability of conformer I, only conformers II and III shown in Figure S5 are able to undergo self-assembly. Therefore, conformers II and III were used to build up the most plausible supramolecular pentamers, which were fully optimized through the cost-effective GFN2-xTB method. Pentamers **1A**_**5**_ and **1B**_**5**_ in parts a and b of Figure 3, respectively, correspond to the most stable aggregates found and show a π-stacking of the aromatic PBI cores with an *anti* disposition of the peripheral benzoate groups. The lowest-energy aggregate **1A**_**5**_ grows up with a shift of approximately 2.90 Å along the long axis of the central N-annulated PBI, and an intermolecular distance of 3.20 Å between adjacent monomers. This aggregate can be initially considered as a typical J-type self-assembly. In contrast, aggregate **1B**_**5**_, which is calculated 23.4 kJ mol^–1^ higher in energy compared to **1A**_**5**_ (GFN2-xTB level in gas phase), self-assembles with a rotational angle (θ) of 35.0°, a growing axis shifted approximately 2.90 Å with respect to the center of the PBI core, and an intermolecular core-to-core distance of 3.25 Å. The stabilizing noncovalent interactions present in both structures are the π-stacking of the central aromatic moieties, as evidenced by the ^1^H NMR spectra shown in Figure S1, and between the peripheral benzoate groups of vicinal molecules, with CH···O contacts of 2.40 Å (**1A**_**5**_) and 2.73–2.94 Å (**1B**_**5**_), and the C=O dipole–dipole electrostatic interactions between vicinal benzoates. From now on, we will focus on the most stable **1A**_**5**_ aggregate, which grows up as a staircase-like aggregate with a negligible helical long-axis-displaced supramolecular structure (Figure S6).

The self-assembled model **1A**_**5**_ was used to further shed light on the cooperative SP mechanism experimentally observed for compounds **1**. Thus, the binding energy per interacting pair (Δ*E*_bind,–-1_), computed at the GFN2-xTB level for **1A**_**5**_-based oligomers in *n*-hexane as solvent and as a function of the oligomer size *n*, displays a rapid decay from the dimer (−76.2 kJ mol^–1^) to the decamer (−91.5 kJ mol^–1^) (Figure S7). After *n* = 10, the decrease in Δ*E*_bind,*n*–1_ is attenuated, and Δ*E*_bind,*n*–1_ converges to a value of ca. −94 kJ/mol when *n* = ∞. This trend in Δ*E*_bind,*n*–1_ confirms a nucleation–elongation SP mechanism, where the initial fast decay is associated with the nucleation and the attenuated part is related to the elongation process in which the addition of extra molecules has no significant effect on the stabilization energy of the aggregate. The presence of different noncovalent interactions stabilizing the self-assembly (especially π-stacking interactions along with oriented C=O dipole–dipole forces) may explain the cooperativity of the supramolecular growth mechanism in **1**.

The optical properties of **1** were studied by means of time-dependent density functional theory (TD-DFT) calculations along with a vibronic excitonic model, similar to that proposed by Spano and co-workers, to deal with the optical properties in the aggregated state (see the Supporting Information for full details).^20^ Experimentally, the UV–vis spectrum of the molecularly dissolved state of **1** shows two strong bands at 485 and 519 nm (Figure 2a). These bands are attributed to the vibrational structure (A_0–1_/A_0–0_ vibronic transitions) associated with the lowest-lying S_0_ → S_1_ electronic excitation according to the simulated absorption spectrum (including vibrational resolution) calculated for a core model of **1** in its monomeric form (Figure S8). The theoretical spectrum predicts two strong bands at 460 and 500 nm, in good agreement with the experimental spectrum, and confirms that the absorption features of **1** mainly result from the N-annulated PBI core. The absorption spectrum of the aggregate was theoretically calculated by using an excitonic model including not only local excitations (Frenkel-type states) but also charge-transfer (CT) states. The latter have been demonstrated to be relevant for the optical properties of PBI-based self-assemblies.^21,22^Figure S8 shows the simulated absorption spectrum for an ideal **1A**_**10**_ aggregate. The spectrum exhibits two well-separated absorption bands (peaking at ca. 430 and 600 nm) accompanied by their corresponding vibrational structure. These spectral features are characteristic of a CT-mediated J-type aggregate and explain the broad and vibrationally resolved experimental band observed in the 400–650 nm range (Figure 2a).^20,21^

The situation changes drastically in compounds **2**, in which the peripheral trialkoxyphenyl units are directly attached to the N-annulated PBI core (Figure 1a). Similarly to compounds **1**, concentration-dependent ^1^H NMR spectra of **2** display the downfield shift of the three sets of aromatic resonances upon decreasing the concentration with a minor shift of the aliphatic protons, indicating the π-stacking of the aromatic units (Figure S9). To unveil the arrangement of the monomeric units of compounds **2** in forming the corresponding supramolecular polymers and the mechanism governing their SP, VT-UV–vis experiments were first performed in MCH as solvent. Unlike compounds **1**, the absorption pattern of achiral **2** in MCH (*c*_T_ = 10 μM, 20 °C) shows a broad band centered at 494 nm with shoulders at 463 and 537 nm, apparently characteristic of H-type aggregates (Figure S10a).^18^ Heating this solution to 90 °C changes the absorption profile, and bands at 517 and 485 nm, ascribable to the monomeric state of the N-annulated PBI core, are observed. However, the intensity ratio between these two bands does not correspond to that recorded in “good” solvents such as CHCl_3_ and toluene (Tol), in which the fully molecularly dissolved state is achieved (Figure S10b). In fact, plotting the variation of the absorption at 517 nm versus temperature results in an incomplete cooling curve that resembles a sigmoidal shape, thus indicating an isodesmic mechanism (Figure S10c).^4^ This incomplete disassembly of the supramolecular polymers of **2** in MCH is also observed by decreasing *c*_T_ to 5 μM (Figure S11) or for the chiral congener (*S*)**-2** (Figure S12a).

**Figure 4:**
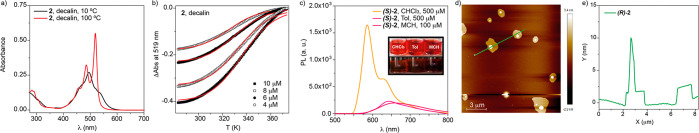
(a) UV–vis spectra of **2** at different temperatures (decalin, *c*_T_ = 10 μM). (b) Plot of the variation of the absorbance measured at 519 nm versus cooling at 1 °C min^–1^. Red curves correspond to the fitting to the EQ model. (c) PL spectra of **(*S*)-2** in MCH, toluene, and CHCl_3_ (λ_exc_ = 380 nm, 298 K). The inset shows pictures of the solutions of **(*S*)-2** in MCH, Tol, and CHCl_3_ used to register the PL spectra without (top samples) and with illumination (bottom samples). (d) AFM image and (e) height profile along the green line in (d) of the supramolecular polymers formed by **(*R*)-2** in MCH onto HOPG (spin coating; *c*_T_ = 10 μM, 298 K).

To achieve a complete disassembly of the aggregates of N-annulated PBIs **2** that allows us to elucidate the SP mechanism and to infer the corresponding thermodynamic parameters, we used decalin as the solvent due to its higher boiling point in comparison to MCH. The UV–vis spectrum of the aggregated species of **2** in decalin presents an absorption profile identical with that registered in MCH (Figure 4a). However, on heating to 100 °C, it is possible to achieve a complete disassembly, as indicated by the relative intensity of the characteristic A_0–1_/A_0–0_ vibronic transitions (Figure 4a). In good analogy with compounds **1**, no thermal hysteresis is observed in the cooling and heating curves, which indicates that the supramolecular polymerization of **2** takes place under thermodynamic control (Figure S12b). Plotting the variation of the absorbance at 519 nm versus temperature results in sigmoidal curves that, upon global fitting to the one-component EQ model, yields the thermodynamic parameters associated with the isodesmic formation of the H-type aggregates (Figure 4b, Figure S13, and Table 1). It is worth noting that, despite the lower values of Δ*H*_e_ for compounds **2** in comparison to those derived for compounds **1** and the similar entropies for both **1** and **2**, the binding constants for these H-type aggregates are one order of magnitude higher than those calculated for the J-type aggregates formed by compounds **1** (Table 1). This difference in the stability of the aggregates is justified by the nucleation penalty present in the cooperative formation of the head-to-tail supramolecular polymers of **1**. The face-to-face arrangement of the self-assembling units of compounds **2** in the H-type aggregates is also confirmed by the strong ACQ effect observed in the fluorescence spectra (Figure 4c). In good analogy to compounds **1**, N-annulated PBIs **2** show an emission spectrum in CHCl_3_ featuring two bands at 586 and 630 nm with a Stokes shift of 53 nm (Figure 4c). In contrast, the emission spectrum of the supramolecular polymers of **2** in MCH shows an unstructured, broad band of very low intensity centered at 662 nm and a large Stokes shift (∼160 nm) (Figure 4c).

The morphology of the H-type aggregates formed from achiral **2** was visualized by AFM. The AFM images of **2** show the formation of 2D nanosheets with diameters of a few micrometers and uniform heights of ∼1.6 nm. On top of these 2D nanosheets, the presence of high micellar aggregates with ∼450 nm thickness and ∼8.5 nm height is also observed (Figure 4d,e and Figure S14).

**Figure 5:**
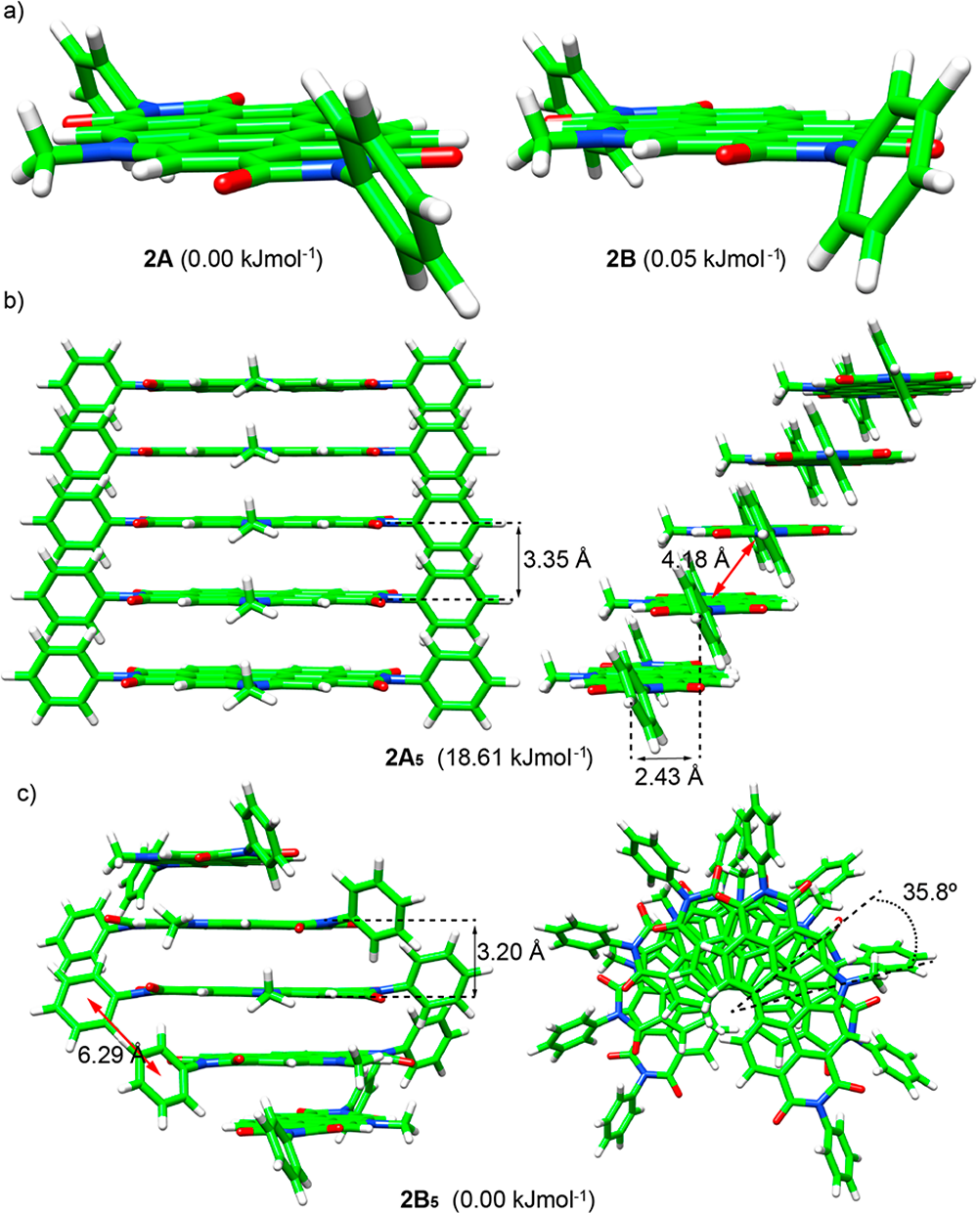
Minimum-energy structures (with their relative energies indicated) calculated at the GFN2-xTB level for the most stable monomers **2A** and **2B** (a) and for pentamers **2A**_**5**_ (b) and **2B**_**5**_ (c) in the self-assembly of **2**.

To justify the sharp differences found in the supramolecular polymerization of compounds **2** in comparison to compounds **1**, the monomeric and aggregated states of **2** were theoretically investigated at the GFN2-xTB level.^19^ As was previously done for compounds **1**, the peripheral alkoxy chains present in the chemical structure of **2** were removed to reduce the computational cost. Due to the more rigid structure of **2**, only two possible conformers, differing in the eclipsed (**2A**) or staggered (**2B**) disposition of the peripheral benzene rings with respect to the long PBI axis, are possible and they were computed to be practically isoenergetic (Figure 5a). Similarly to **1**, the supramolecular pentamers **2A**_**5**_ and **2B**_**5**_ were modeled and were fully optimized at the GFN2-xTB level (Figure 5b). In both aggregates, the pyrrolic units of the central PBI cores are arranged in the most favorable parallel distribution along the stacking axis, as demonstrated previously.^14^ The helical aggregate **2B**_**5**_, which stems from monomers **2B** with the peripheral aromatic groups in a staggered disposition, was calculated to be more stable by a small energy difference of 18.61 kJ mol^–1^. **2B**_**5**_ grows with a rotational angle (θ) of 35.8° along a stacking axis that is shifted ca. 2.90 Å with respect to the center of the perylene scaffold. Adjacent molecules in **2B**_**5**_ are separated by an intermolecular distance of 3.20 Å between the aromatic cores and are arranged with the pyrrole moieties pointing out of the stack. In contrast, aggregate **2A**_**5**_, built up from monomers **2A** displaying an eclipsed conformation of the phenyl groups, grows in a slipped manner with a shift of 2.43 Å along the short PBI axis and an intermolecular distance of 3.35 Å between consecutive aromatic cores. In both structures, stabilizing noncovalent intermolecular interactions due to the π-stacking of both the central PBI cores and the peripheral phenyl groups and to the CH(phenyl)···O interactions (distances of 2.02 Å in **2A**_**5**_ and 2.25–2.48 Å in **2B**_**5**_) are present (Figure 5b). Pentamers **2A**_**5**_ and **2B**_**5**_ can both be initially considered as H-type aggregates.

To get further insight into the spectroscopic features displayed by compounds **2**, TD-DFT theoretical calculations and the excitonic model mentioned above were used to compute the UV–vis spectra. Figure S15 displays the simulated absorption spectra for ideal **2A**_**10**_ and **2B**_**10**_ decamers. A broad vibrationally resolved absorption band is obtained for both aggregates, although with different spectral profiles. The pattern predicted for **2B**_**10**_ is especially in good accord with that experimentally registered in decalin for the supramolecular polymers formed from compounds **2** (Figure 4a) and confirms the H-type nature of this aggregate. It is important to note that, as discussed below, **2B** supramolecular oligomers are more stable than **2A** oligomers in apolar solvents.

### Chiroptical Features: Transfer and Amplification of Asymmetry.^23^

The decoration of compounds **(*S*)-1**, **(*R*)-1**, **(*S*)-2**, and **(*R*)-2** with chiral side chains, together with the studies on the supramolecular polymerization of these N-annulated PBIs discussed above, allows performing a detailed investigation on the transfer and amplification of asymmetry resulting from these self-assembling units.

**Figure 6:**
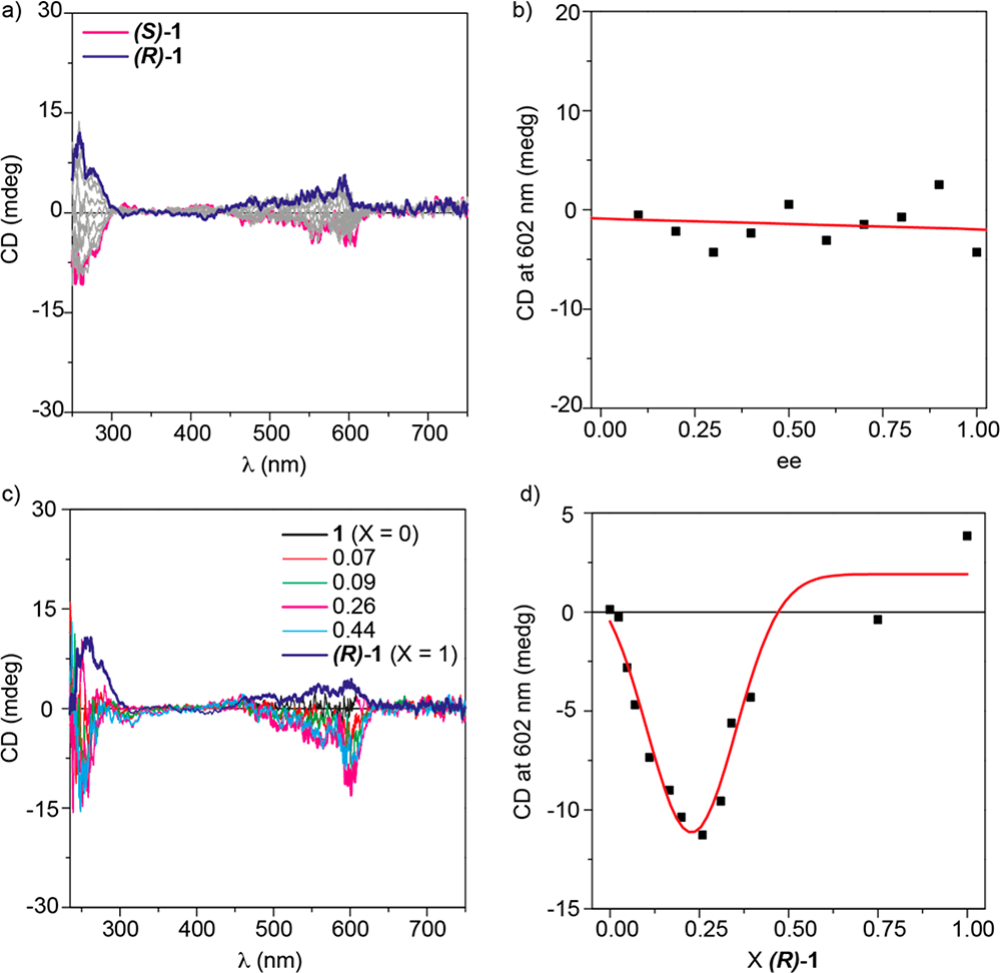
Amplification-of-asymmetry experiments performed with N-annulated PBIs **1**. (a) ECD spectra for MR experiments. (b) Changes in ECD intensity at 602 nm as a function of the ee upon adding **(*R*)-1** to a solution of **(*S*)-1**. (c) Selected ECD spectra registered for the SaS experiments performed by mixing **1** and **(*R*)-1**. (d) Changes in the ECD intensity at 602 nm upon increasing the molar fraction (X) of the chiral sergeant (*R*)**-1**. The red line in (d) corresponds to a Gaussian fit to guide the eye. Experimental conditions: MCH, *c*_T_ = 50 μM, 20 °C.

The cooperative supramolecular polymerization found for compounds **1** was expected to yield an efficient transfer of asymmetry from the molecular to the supramolecular level. Previous examples of J-type aggregates have been reported to give rise to helical aggregates, as demonstrated by the corresponding ECD spectra.^24^ To our surprise, the ECD spectra of the J-type aggregates formed by **(*S*)-1** and **(*R*)-1** in MCH display a very weak and noisy dichroic response with maxima at 260 and 591 nm and the anisotropy factor *g* ≈ 1 × 10^–4^ (Figure 6a). The negligible chiroptical response observed for compounds **1** is in agreement with the nonhelical long-axis-displaced supramolecular structure theoretically predicted for the aggregates derived from **1** (Figure 3a). The lack of dichroic response is also corroborated by the noisy VCD spectra recorded for **(*S*)-1** and **(*R*)-1,** which contrasts with the well-defined FTIR spectra of these N-annulated PBIs in solution, where the bands corresponding to the imide and ester carbonyls (1714 and 1692 cm^–1^) and to the aryl alkyl ethers (1218 and 1118 cm^–1^) are clearly visible (Figure S16). In addition, and despite the strong emission of the supramolecular polymers formed by these chiral N-annulated PBIs (Figure 2c), no CPL signal is observed (Figure S17a).

We also evaluated the ability of chiral N-annulated PBIs **1** to undergo amplification of asymmetry by developing majority rules (MR) and sergeants-and-soldiers (SaS) experiments.^11a,12^ In the MR experiments, unequal amounts of the two enantiomers **(*S*)-1** and **(*R*)-1**, with the total concentration kept constant, are mixed together. In this case, the ECD spectra registered at different **(*S*)-1**/**(*R*)-1** ratios show the same weak and noisy profile as that registered for pristine enantiomers (Figure 6a). In addition, plotting the variation of the poor dichroic response at 602 nm versus the enantiomeric excess (ee) results in a linear trend, which indicates either the negligible ability of these compounds to coassemble into homochiral aggregates or the inability of detecting this phenomenon due to the poor dichroic response (Figure 6b). The lack of a clear dichroic (ECD, VCD, CPL) response for the supramolecular polymers formed by **(*S*)-1** and **(*R*)-1**, despite the cooperative character of their supramolecular polymerization, is accounted for by the staircaselike aggregation mode (Figures 1a and 3a and Figure S6) that yields a nonhelical supramolecular structure.

The SaS experiments, performed by mixing the achiral soldier **1** and the chiral sergeant **(*R*)*-*1**, produce an abnormal effect by which the sign of the dichroic signal detected for relatively low molar fractions (X) of **(*R*)*-*1** is reversed with respect to that recorded for the pristine chiral seargent (Figure 6c,d). The weak dichroic response attained in the ECD spectra could suggest that this effect is an artifact. However, an analogous effect but of opposite sign is observed for the SaS experiments carried out by mixing **1** and **(*S*)*-*1** (Figure S18). We have previously described a similar effect for self-assembling units able to form intramolecular hydrogen-bonded metastable monomers that retard the corresponding supramolecular polymerization.^25^ However, this is not the case, since N-annulated PBIs **1** cannot form H-bonded metastable monomers and their supramolecular polymerization is under thermodynamic control. A similar abnormal SaS effect has been described for covalent polymers and justified by considering the different energy preferences exhibited by the situation in which a chiral sergeant is adjacent to another chiral sergeant or to an achiral soldier. This energy difference conditions the sign of the dichroic response.^26^

**Figure 7:**
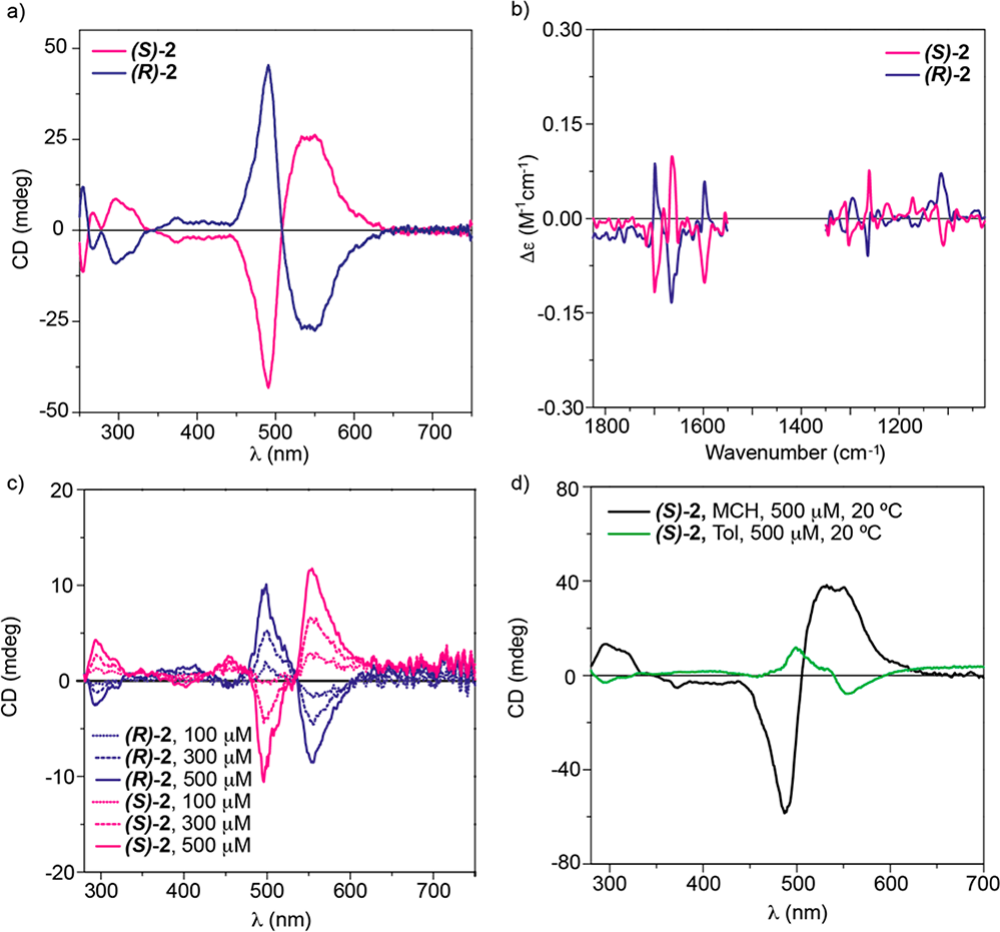
(a) ECD spectra of **(*S*)-2** and **(*R*)-2** in MCH (*c*_T_ = 10 μM, 20 °C). (b) VCD spectra of **(*S*)-2** and **(*R*)-2** in MCH (*c*_T_ = 0.02–0.03 M, 20 °C). (c) ECD spectra of **(*S*)-2** and **(*R*)-2** in Tol at 20 °C. (d) ECD spectra of **(*S*)-2** and **(*R*)-2** in MCH and Tol (*c*_T_ = 500 μM, 20 °C).

In contrast to **1**, the transfer of asymmetry from the molecular to the supramolecular level in N-annulated PBIs **(*S*)-2** and **(*R*)-2** yields a rich dichroic pattern, with an intense bisignated Cotton effect with maxima at 490 and 547 nm and zero crossing points at 508, 344, 278, and 261 nm and with the anisotropy factor *g* ≈ 3 × 10^–3^ (Figure 7a). This bisignated Cotton effect implies the formation of *P-* and *M-*type helical aggregates for **(*S*)-2** and **(*R*)-2**, respectively.^27^ The formation of helical aggregates from **(*S*)-2** and **(*R*)-2** is in agreement with the helical structure predicted theoretically (Figure 5c) and is corroborated by the corresponding VCD spectra that display a mirror-like vibrational pattern (Figure 7b). VCD spectroscopy has indeed been recognized as a suitable chiroptical tool to characterize helical supramolecular polymers.^28^ This mirror-like vibrational pattern is observed for those bands at 1700, 1665, and 1600 cm^–1^, ascribable to the conjugated aromatic units, and at 1233 and 1111 cm^–1^, corresponding to the stretching of the alkoxy groups (Figure 7b and Figure S19). On the other hand, the strong ACQ effect observed in the corresponding fluorescence measurements (Figure 4c) results in null CPL emission from the aggregates formed by **(*S*)-2** and **(*R*)-2** (Figure S17b).

Taking into account the efficient transfer of asymmetry experienced by **(*S*)-2** and **(*R*)-2**, we checked the ability of these N-annulated PBIs to amplify asymmetry by developing MR experiments. A linear increase in the dichroic response is observed upon plotting the variation of the ECD signal versus the ee, diagnostic of the lack of amplification of asymmetry (Figure S20). Similar findings were inferred from the corresponding SaS experiments performed by mixing **2** and **(*S*)-2**, for which a linear increase of the dichroic response upon increasing the amount of the chiral sergeant was observed (Figure S21). These studies reveal the ability of *N*-PBIs **2**, which form supramolecular polymers governed by an isodesmic mechanism, to undergo an efficient transfer of asymmetry but a negligible amplification of asymmetry.

Finally, the influence of the solvent on the chiroptical features of the reported N-annulated PBIs was evaluated. The solvent–solute interactions are crucial in tuning the solubility, reactivity, and structure of organic compounds and are at the forefront of the research on supramolecular polymers, since the solvent can efficiently bias the assembly/disassembly process.^29^ Some reports describe that the chirality embedded in solvent molecules exerts a pivotal role in controlling the helical outcome resulting from the supramolecular polymerization of achiral or chiral self-assembling units.^30^ However, there are scarce examples in the literature of solvent-controlled stereomutation in supramolecular polymers formed under thermodynamic control and directed by molecular design.^31^ In our case, we envision that the two conformers of the monomeric species of **2**, which are isoenergetic (Figure 5a), could originate helical or linear (with a small net helicity) aggregates of opposite handedness depending on the solvent conditions. To investigate this potential stereomutation, we first recomputed the stability of the optimized pentamers **2A**_**5**_ and **2B**_**5**_ by including *n*-hexane (as a representative solvent that emulates the MCH nature) or toluene as solvent. Frequency calculations were additionally carried out to estimate the free energy of the aggregation process. The self-assembly free energies per interacting pair (Δ*G*_bind,*n*–1_^solv^) in *n*-hexane and toluene are gathered in Table 2. In *n*-hexane, the relative stability calculated in the gas phase is preserved, and the helical **2B**_**5**_ aggregate is more stable than the linear **2A**_**5**_ aggregate by 8.30 kJ mol^–1^. In contrast, the stability is reversed in Tol and the linear aggregate is slightly more stable by 0.59 kJ mol^–1^. The relative stability of the aggregates of compound **2** therefore depends on the solvent nature and, when the most stable aggregate in each solvent is considered, Δ*G*_bind,*n*–1_^*n*-hexane^ is calculated to be more negative than Δ*G*_bind,*n*–1_^toluene^ by ∼3.0 kJ mol^–1^. These results thus point to a higher tendency of compounds **2** to self-assemble in MCH than in Tol, in good accord with the experimental evidence.

**Table 2:** Self-Assembly Free Energy (Δ*G*_bind,*n*–1_^solv^, in kJ mol^–1^) Estimated for the Two Most Stable Aggregates of **2** in *n*-Hexane and Toluene Solution at the GFN2-xTB/GBSA Level

aggregate	Δ*G*_bind,*n*–1_^*n*–hexane^	Δ*G*_bind,*n*–1_^toluene^
**2A_5_**	–19.63	–24.94
**2B_5_**	–27.93	–24.35

The supramolecular mechanism of the self-assembling process of N-annulated PBIs **2** was furthermore investigated by performing interaction energy calculations for regular oligomers of increasing size (from *n* = 1 to *n* = 50 monomers) at the GFN2-xTB level in the presence of the solvent. Theoretical calculations on **2A**_**5**_-type aggregates in toluene reveal that the binding energy per interacting pair (Δ*E*_bind,*n*–1_) barely changes with the oligomer size (Figure S22), which is a clear indication of an isodesmic supramolecular polymerization mechanism. Similar results are obtained for **2B**_**5**_-type oligomers in *n*-hexane (Figure S22), with even a small Δ*E*_bind,*n*–1_ increase of ∼5 kJ mol^–1^ from *n* = 2 to *n* = 5 in this case. The absence of cooperativity predicted in the supramolecular growth of **2** stems from the nature of the noncovalent interactions stabilizing the self-assembly (mainly π–stacking interactions), with no directional electrostatic forces such as H-bonding or dipole–dipole interactions.^32^

Taking into account the previous report on the stereomutation experienced by a comparable PBI^33^ and the results of the theoretical calculations, we experimentally checked the possibility of **(*S*)-2** and **(*R*)-2** of achieving a solvent-controlled stereomutation by registering the ECD spectra in Tol as solvent. Using concentrations as low as those utilized in MCH (Figure 7a), no ECD response was observed. However, when the concentration was increased to *c*_T_ = 100 μM, it was possible to register a weak but noticeable ECD spectrum (Figure 7c), showing patterns opposite to those recorded in MCH (Figure 7a). It is worth noting that the UV–vis spectrum recorded at *c*_T_ = 100 μM reveals a degree of aggregation that increases upon increasing the concentration up to 500 μM, as the higher intensity observed in the dichroic response demonstrates (Figure 7c and Figure S23). At *c*_T_ = 500 μM, the dichroic pattern of N-annulated PBIs **2** in Tol is opposite to that registered in MCH (Figure 7d) and the sample is completely aggregated, as supported by the quenching of the fluorescence emission (Figure 4c).

The influence of the type of aggregation on the circular dichroism properties of N-annulated PBIs was also analyzed by means of TD-DFT calculations. To unveil the effect of the rotational dihedral angle (θ) along the growth axis, the theoretical ECD spectrum was calculated for a dimer of the N-annulated PBI core, in which the angle θ ranges from 0 to 10° (Figure S24). Theoretical calculations indicate that a significant dichroic signal is obtained even at small dihedral angles (>2°). This suggests that not only helical aggregates with a large θ value but also linear stacking aggregates with a small net θ value, promoted by the point chirality element embedded in the peripheral groups, may afford a dichroic signal. The different dichroic reponses observed for chiral **2** in MCH and Tol can thus be explained by the different aggregation modes of **2** in these two solvents. In toluene, theoretical calculations predict that the most stable aggregate **2A**_**5**_ is an almost regular linear H-type self-assembly and gives rise to a small dichroic signal (Figure S25) in agreement with the experiment (Figure 7c,d). In contrast, in *n*-hexane (modeling MCH), the most stable aggregate is predicted to be **2B**_**5**_, which corresponds to a well-defined helical arrangement with a rotational dihedral angle θ of 35° and thus shows an intense dichroic pattern (Figure S25) similar to that found experimentally for **(*S*)-2** and **(*R*)-2** in MCH (Figure 7c,d).

## Conclusions

The synthesis of two series of N-annulated PBIs endowed with peripheral trialkoxyphenyl groups directly attached to the imide nitrogens of the PBI core (**2**) or linked by a propionate spacer (**1**) is reported. A complete set of spectroscopic measurements and theoretical calculations demonstrate the huge influence that the distance and conformational flexibility of the peripheral groups exert on the optical and chiroptical properties and on the supramolecular polymerization mechanism of the resulting self-assembly. Compounds **1** present a cooperative supramolecular polymerization that yields highly emissive J-type aggregates with negligible chiroptical (ECD, VCD, and CPL) response for the chiral congeners **(*S*)-1** and **(*R*)-1**. The staircase-like aggregation mode calculated for the supramolecular polymers formed from **1** and the dipole–dipole interactions between adjacent C=O groups along the stack justify these features. In contrast, bringing the peripheral side groups closer to the N-annulated PBI core drastically changes the self-assembling features of compounds **2**. In this case, the supramolecular polymerization is governed by an isodesmic mechanism that gives rise to H-type aggregates exhibiting a strong ACQ effect which, therefore, show low emissive properties. Chiral **(*S*)-2** and **(*R*)-2** experience an efficient transfer of asymmetry to afford *P-* and *M-*type aggregates, respectively, but no amplification of asymmetry is achieved by performing MR and SaS experiments. Finally, a solvent-controlled stereomutation has been demonstrated for chiral **(*S*)-2** and **(*R*)-2**, which form supramolecular polymers with different structures depending on the solvent utilized (MCH or Tol). This stereomutation has been accounted for by considering the two possible conformations of the peripheral side chains in compounds **2**, eclipsed or staggered. These conformations lead to linear or helical self-assemblies, respectively, of opposite stability depending on the solvent conditions. The synergy between the experimental evidence and the theoretical calculations presented herein contribute to elaborate structure/function relationships useful in predicting relevant features of supramolecular polymers.

## Supporting Information Available

Full experimental details and characterization, additional spectroscopic measurements, and theoretical calculations, including Figures S1–S25 (PDF)

## Abbreviations

PBI: perylene bisimide
ACQ: aggregation-caused quenching
SaS: sergeants and soldiers
MR: majority rules
SP: supramolecular polymerization
CT: charge transfer
ECD: electronic circular dichroism
VCD: vibrational circular dichroism
MCH: methylcyclohexane
AFM: atomic force microscopy
HOPG: highly oriented pyrolytic graphite
EQ: equilibrium
GBSA: generalized Born and surface area continuum solvation model.
