# Supplementary material for: Distance Matters: Biasing Mechanism, Transfer of Asymmetry, and Stereomutation in N-Annulated Perylene Bisimide Supramolecular Polymers
Source: J Am Chem Soc. 2021 Aug 11;143(33):13281–91. doi: 10.1021/jacs.1c06125 (PMC8478275; doi:10.1021/jacs.1c06125)
Supplement: Supplementary file 1 — ja1c06125_si_001.pdf [file ja1c06125_si_001.pdf]

Supporting Information

Distance Matters: Biasing Mechanism, Transfer of Asymmetry, and Stereomutation in *N*-Annulated Perylene Bisimide Supramolecular Polymers

Manuel A. Martínez,[a] Azahara Doncel-Giménez,[b] Jesús Cerdá,[b] Joaquín Calbo,[b] Rafael Rodríguez,[c] Juan Aragó,[b] Jeanne Crassous,[c] Enrique Ortí,*,[b] and Luis Sánchez*,[a]

[a] *Departamento de Química Orgánica, Facultad de Ciencias Químicas, Universidad Complutense de Madrid, E-28040 Madrid, Spain.*

[b] *Instituto de Ciencia Molecular (ICMol), Universidad de Valencia, c/Catedrático José Beltrán, 2, E-46980 Paterna, Spain.*

[c] *Univ Rennes, CNRS, ISCR (Institut des Sciences Chimiques de Rennes) – UMR 6226, F-35000 Rennes, France.*

Contents:

1. Experimental section S-2
2. Synthetic details and characterization S-3
3. Collection of spectra S-7
4.- Supplementary Figures S-15
Concentration dependent ^{1}*H NMR spectra of* ***(S)-1*** S-15
UV-Vis spectra of ***(S)-1*** S-15
Photophysical Parameters for compounds ***(S)-1*** *and* ***(S)-2*** S-16
AFM images of ***(S)-1*** S-16
Optimized geometries and modelized UV-Vis for ***1*** S-16
Concentration dependent ^{1}*H NMR spectra of* ***2*** S-18
UV-Vis spectra of compounds ***2*** S-18
AFM images of ***(S)-2*** S-19
Simulated absorption spectra S-20
FTIR and VCD spectra of compounds ***(S)-1*** *and* ***(R)-1*** S-20
Emission and CPL spectra S-20
SaS experiments of compounds ***1*** *and* ***(S)-1*** S-21
FTIR spectra of compounds ***(S)-2*** *and* ***(R)-2*** S-21
MRs and SaS experiments of compounds ***2*** S-21
Solvent dependent optimized energy changes for compounds ***2*** S-22
UV-Vis spectra of compound ***(S)-2*** *in toluene* S-22
Simulated ECD spectra S-23
5. Theoretical Calculations S-24
6. References S-29

1. Experimental section

General. All solvents were dried according to standard procedures. Reagents were used as purchased. All air-sensitive reactions were carried out under argon atmosphere. Flash chromatography was performed using silica gel (Merck, Kieselgel 60, 230–240 mesh or Scharlau 60, 230–240 mesh). Analytical thin layer chromatography (TLC) was performed using aluminium-coated Merck Kieselgel 60 F254 plates. NMR spectra were recorded on a Bruker Avance 300 MHz (1H: 300 MHz; 13C: 75 MHz) spectrometer at 298 K using partially deuterated solvents as internal standards. Coupling constants (*J*) are quoted in Hz and chemical shifts (δ) in ppm. Multiplicities are denoted as follows: s = singlet, d = doublet, t = triplet, m = multiplet, br = broad. FT-IR spectra were recorded on a Bruker Tensor 27 (ATR device) spectrometer. UV-Vis spectra were registered on a Jasco-V630 spectrophotometer equipped with a Peltier thermoelectric temperature controller. Electronic circular dichroism (ECD) measurements were performed in a Jasco-J1500 spectrophotometer equipped with a Peltier thermoelectric temperature controller (Jasco MCB-100 model). The spectra were recorded in the continuous mode between 750 and 220 nm, with a wavelength increment of 0.2 nm, a response time of 1 s, and a bandwidth of 2 nm. 1 cm and a 1 mm path-length quartz cuvettes (Hellma) with screw cap were used. Atomic force microscopy was performed on a SPM Nanoscope IIIa multimode microscope working on tapping mode with a TESPSS tip (Veeco) at a working frequency of ~235 kHz. High resolution mass spectra (HRMS) were recorded on a MALDI Bruker daltonics Ultraflex TOF/TOF spectrometer. IR and VCD spectra were recorded on a Jasco FSV-6000 spectrometer in a 200 microns cell and at concentrations of 0.02–0.03 M. Emission spectra was recorded in a Jasco FP8300. Circularly polarized luminescence (CPL) measurements were performed using a home-built (with the help of the JASCO company) CPL spectrofluoropolarimeter. The samples were excited using a 90° geometry with a Xenon ozone-free lamp 150 W LS.

The thermodynamic parameters associated to the supramolecular polymerization mechanism werw derived by applying the Matlab® script built up to execute the one-component equilibrium model published by ten Eikelder and coworkers.[S1] To attain accurate values for the thermodynamic parameters, global fittings using four cooling curves, corresponding to four different concentrations of the monomer species, were performed.

The emission quantum yields and lifetime were measured on the ScanMat Platform (UMS 2001) in Rennes. The emission quantum yield was estimated by using a Hamamatsu C9920–03 apparatus consisting of a 150 W Xenon lamp, a monochromator, an integrating sphere, and a PMA-12 detector (350-1100 nm), using 4 mL quartz cuvettes. The decays were calculated by using an Ocean optics QEPro CCD detector (Range: 350-1100 nm). The excitation source has been a picosecond laser diode (10 KHz to 100 MHz) at 375 nm capable of operating in Burst mode below 10 KHz. For the detection windows, signals in UV-Vis (350-950 nm) are recorded as a function of time over a range of 135 nm simultaneously using a Hamamatsu C10910-25 streak camera mounted with a slow single sweep drawer. The range of measurable lifetimes is 100-300 μs.

2. Synthetic details and characterization

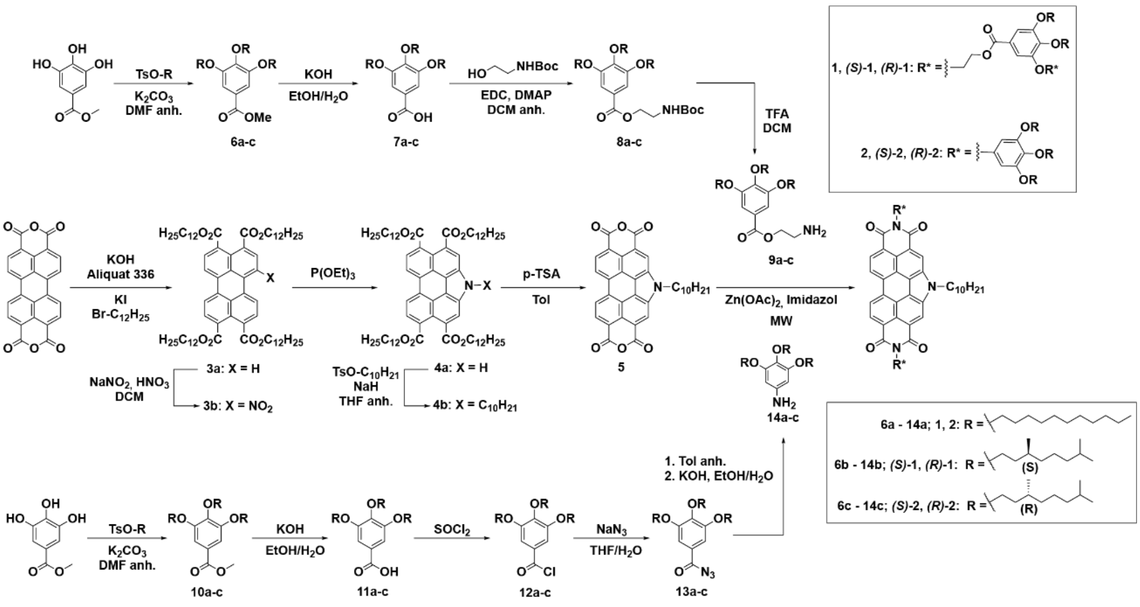

Scheme S1. Synthesis of the reported *N*-annulated PBIs **1** and **2**.

Compounds **3-14** and compound **1** were prepared according to previously reported synthetic procedures and showed identical spectroscopic properties to those reported therein.[S2-S4]

Synthesis of PBIs 1 and 2. General procedure.

Compound **5** (38 mg, 0.07 mmol), amines **9** or **14** (0.15 mmol), zinc acetate (13 mg, 0.07 mmol), and 460 mg of imidazole were introduced in a 10 mL microwave tube. After three cycles of argon/vacuum, the compound mixture was reacted in a microwave (165 °C, 35 minutes). The crude was redissolved in CH_2Cl_2 and washed with brine. Organic layers were dried over $MgSO_4$ and the solvent was evaporated under reduced pressure. The residue was purified by column chromatography (CH_2Cl_2/MeOH 10/0.1).

(*S,S,S*)-(5-decyl-1,3,7,9-tetraoxo-1*H*-pyrido[3',4',5':4,5]naphtho[2,1,8-*cde*]pyrido[3',4',5':4,5]naphtho[8,1,2-*ghi*]isoindole-2,8(3*H*,5*H*,7*H*,9*H*)-diyl)bis(ethane-2,1-diyl)bis(3,4,5-tris(((*S*)-3,7-dimethyloctyl)oxy)benzoate) [*(S)*-1]

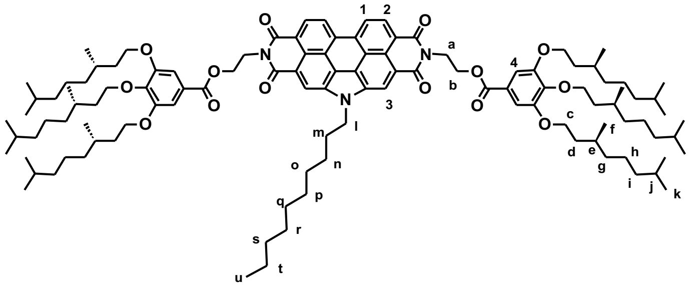

$C_{112}H_{165}N_3O_{14}$
Exact Mass: 1776.2292
Mol. Wt.: 1777.5202

Compound ***(S)*-1** was obtained as a bright red solid (57 mg, 25 %). ^{1}H-NMR ($CDCl_3$, 300 MHz, 323 K) δ 8.89 (2H, H_3, s), 8.81 (2H, H_1, d, J = 8.1 Hz), 8.74 (2H, H_2, d, J = 8.1 Hz), 7.25 (4H, H_4, s), 4.84 (2H, H_l, t, J = 7.2 Hz), 4.81 (4H, H_b, t, J = 5.1 Hz), 4.72 (4H, H_a, t, J = 5.1 Hz), 4.07–3.91 (12H, H_c, m), 2.21–2.08 (2H, H_m, m), 1.89–1.72 (6H, H_e, m), 1.71–1.40 (24H, H_{d+g}, m), 1.40–1.02 (44H, $H_{(h-j)+(n-t)}$, m), 0.95–0.76 (57H, H_{f+k+u}, m). ^{13}C-NMR ($CDCl_3$, 75 MHz, 298K) δ 166.5, 165.1, 163.9, 152.9, 142.3, 134.9, 132.9, 127.8, 124.7, 124.6, 124.0, 122.2, 121.9, 121.6, 119.6, 119.0, 108.1, 77.4, 71.8, 67.4, 62.7, 39.7, 39.5, 39.4, 37.6, 37.5, 37.4, 36.5, 31.9, 31.8, 30.0, 29.8, 29.6, 29.3, 28.1, 27.4, 24.9, 24.8, 22.8, 22.7(4), 22.7(1), 19.7, 14.2. FT-IR (neat) 2954, 2925, 2868, 1692, 1658, 1598, 1560, 1464, 1430, 1383, 1359, 1318, 1255, 1214, 1117, 1024, 869, 803, 763, 739 cm^{-1}. HRSM (MALDI-TOF, exact mass) calcd. for $C_{112}H_{165}N_3O_{14}$ [M]$^+$, 1776.2292; found, 1801.3782.

(*R,R,R*)-(5-decyl-1,3,7,9-tetraoxo-1*H*-pyrido[3',4',5':4,5]naphtho[2,1,8-*cde*]pyrido[3',4',5':4,5]naphtho[8,1,2-*ghi*]isoindole-2,8(3*H*,5*H*,7*H*,9*H*)-diyl)bis(ethane-2,1-diyl)bis(3,4,5-tris(((*R*)-3,7-dimethyloctyl)oxy)benzoate) [*(R)*-1]

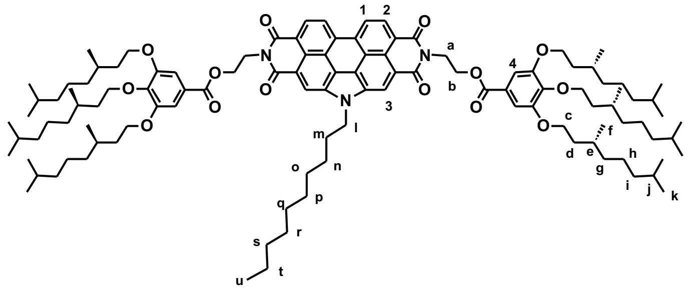

$C_{112}H_{165}N_3O_{14}$
Exact Mass: 1776.2292
Mol. Wt.: 1777.5202

Compound ***(R)*-1** was obtained as a bright red solid (38 mg, 19 %). ^{1}H-NMR ($CDCl_3$, 300 MHz, 323 K) δ 8.89 (2H, H_3, s), 8.82 (2H, H_1, d, J = 8.1 Hz), 8.75 (2H, H_2, d, J = 8.1 Hz), 7.25 (4H, H_4, s), 4.84 (2H, H_l, t, J = 7.0 Hz), 4.81 (4H, H_b, t, J = 5.0 Hz), 4.72 (4H, H_a, t, J = 5.0 Hz), 4.06–3.92 (12H, H_c, m), 2.20–2.09 (2H, H_m, m), 1.89–1.75 (6H, H_e, m), 1.72–1.40 (24H, H_{d+g}, m), 1.40–1.01 (44H, $H_{(h-j)+(n-t)}$, m), 0.91–0.80 (57H, H_{f+k+u}, m). ^{13}C-NMR ($CDCl_3$, 75 MHz, 298K) δ 166.5, 165.1, 164.0, 152.9, 142.3, 134.9, 132.9, 127.8, 124.7, 124.0, 122.2, 121.9, 121.6, 119.6, 119.1, 108.1, 77.4, 71.8, 67.4, 62.8, 39.6, 39.5, 39.4, 37.6, 37.5, 37.4, 36.5, 31.9, 31.8, 30.0, 29.8, 29.6, 29.3, 28.1, 27.4, 24.9, 24.8, 22.8, 22.7(4), 22.7(1), 19.7, 14.2. FT-IR (neat) 2954, 2925, 2869, 1692, 1658, 1599, 1560, 1464, 1430, 1383, 1358, 1318, 1255, 1214, 1107, 1024, 869, 804, 763, 739 cm^{-1}. HRSM (MALDI-TOF, exact mass) calcd. for $C_{112}H_{165}N_3O_{14}$ [M]$^{+}$, 1776.2292; found, 1776,2285.

5-decyl-2,8-bis(3,4,5-tris(dodecyloxy)phenyl)-1*H*-pyrido[3',4',5':4,5]naphtho[2,1,8-*cde*]pyrido [3',4',5':4,5]naphtho[8,1,2-*ghi*]isoindole-1,3,7,9(2*H*,5*H*,8*H*)-tetraone (2)

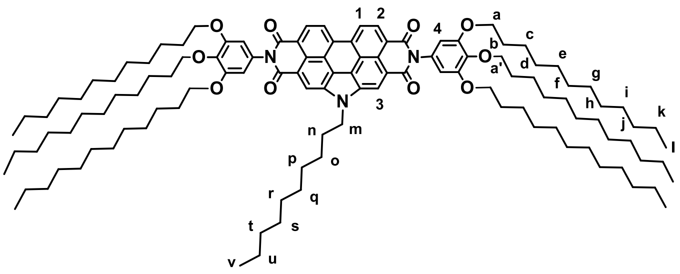

$C_{118}H_{181}N_3O_{10}$
Exact Mass: 1800.3747
Mol. Wt.: 1801.7138

Compound **2** was obtained as a dark red solid (96 mg, 76 %). ^{1}H-NMR ($CDCl_3$, 300 MHz, 323 K) δ 8.76 (2H, H_3, s), 8.71 (2H, H_1, d, J = 8.0 Hz), 8.56 (2H, H_2, d, J = 8.0 Hz), 6.73 (4H, H_4, s), 4.80 (2H, H_m, t, J = 6.1 Hz), 4.07 (4H, $H_{a'}$, t, J = 6.6 Hz), 3.91 (8H, H_a, t, J = 6.6 Hz), 2.12–2.00 (2H, H_n, m), 1.90–1.70 (12H, H_b, m), 1.49–1.11 (122H, $H_{(c-k)+(o-u)}$, m), 0.94–0.76 (21H, H_{l+v}, m). ^{13}C-NMR ($CDCl_3$, 75 MHz, 298K) δ 165.0, 163.7, 153.8, 138.2, 134.2, 132.0, 130.9, 127.5, 123.9, 123.5, 122.4, 121.7, 120.9, 118.6, 118.0, 107.3, 77.4, 73.6, 69.3, 32.1, 32.0, 31.9, 31.7, 30.7, 30.1, 30.0, 29.9, 28.8(9), 28.8(7), 29.7, 29.6(3), 29.6(1), 29.5, 29.4(9), 29.4(2), 29.3, 29.2, 27.2, 26.4, 26.3, 22.9, 22.8, 22.7, 14.3, 14.2, 14.1. FT-IR (neat) 2923, 2854, 1702, 1666, 1602, 1505, 1467, 1438, 1425, 1380, 1305, 1235, 1175, 1116, 804, 740, 711 cm^{-1}. HRSM (MALDI-TOF, exact mass) calcd. for $C_{118}H_{181}N_3O_{10}$ [M+H]$^{+}$, 1801.3747; found, 1801.3782.

5-decyl-2,8-bis(3,4,5-tris(((*S*)-3,7-dimethyloctyl)oxy)phenyl)-1*H*-pyrido[3',4',5':4,5] naphtha[2,1,8-*cde*]pyrido[3',4',5':4,5]naphtho[8,1,2-*ghi*]-isoindole-1,3,7,9(2*H*,5*H*, 8*H*)-tetraone [*(S)*-2]

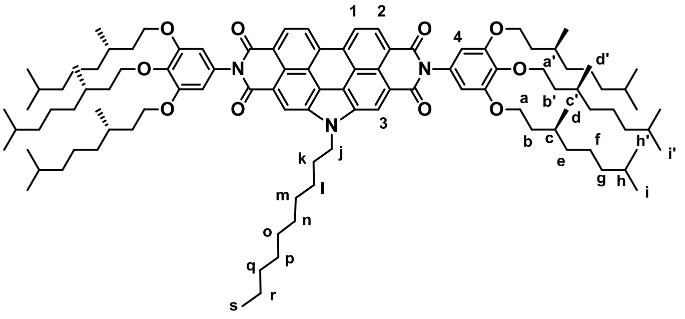

$C_{106}H_{157}N_3O_{10}$
Exact Mass: 1632.1869
Mol. Wt.: 1633.3949

Compound ***(S)*-2** was obtained as a dark red solid (65 mg, 22 %). ^{1}H-NMR ($CDCl_3$, 300 MHz, 323 K) δ 8.83 (2H, H_3, s), 8.77 (2H, H_1, d, J = 8.0 Hz), 8.67 (2H, H_2, d, J =8.0 Hz), 6.72 (4H, H_4, s), 4.83 (2H, H_j, t, J = 6.8 Hz), 4.17–4.03 (4H, $H_{a'}$, m), 3.97 (8H, H_a, t, J = 6.3 Hz), 2.14–2.03 (2H, H_k, m), 2.00–1.74 (8H, $H_{b'+c'+h'}$, m), 1.72–1.45 (16H, H_{b+c+h}, m), 1.43–1.07 (50H, $H_{(e-g)+(l-r)}$, m), 0.99 (6H, $H_{d'}$, d, J = 6.6 Hz), 0.93–0.87 (24H, $H_{d+i'}$, m), 0.85 (24H, H_i, d, J = 6.6 Hz), 0.8 (3H, H_s, t, J = 7.0 Hz). ^{13}C-NMR ($CDCl_3$, 75 MHz, 298K) δ 165.1, 163.8, 153.9, 138.2, 134.3, 132.2, 130.9, 127.6, 124.1, 123.6, 122.4, 121.8, 121.1, 118.8, 118.2, 107.2, 77.4, 71.8, 67.6, 39.6, 39.5, 37.8, 37.7, 37.6(4), 37.6(1), 36.6, 31.9, 31.7, 30.1, 30.0, 29.5, 29.4, 29.3, 29.2, 28.2, 28.1, 27.2, 24.9, 24.8, 22.9, 22.8(6), 22.8(2), 22.7, 22.6, 19.8, 19.6, 14.2. FT-IR (neat) 2955, 2927, 2870, 1702, 1665, 1602, 1504, 1467, 1439, 1424, 1398, 1383, 1366, 1304, 1239, 1174, 1114, 804, 740, 712 cm^{-1}. HRSM (MALDI-TOF, exact mass) calcd. for $C_{106}H_{157}N_3O_{10}$ $[M+H]^+$, 1633.1869; found, 1633.1792.

5-decyl-2,8-bis(3,4,5-tris(((*R*)-3,7-dimethyloctyl)oxy)phenyl)-1*H*-pyrido[3',4',5':4,5] naphtha[2,1,8-*cde*]pyrido[3',4',5':4,5]naphtho[8,1,2-*ghi*]iso-indole-1,3,7,9(2*H*,5*H*, 8*H*)-tetraone [*(R)*-2]

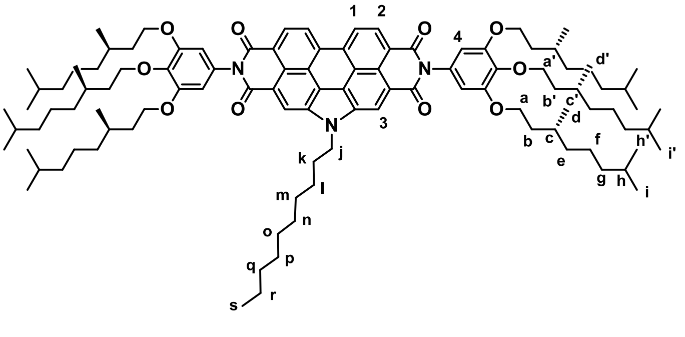

$C_{106}H_{157}N_3O_{10}$
Exact Mass: 1632.1869
Mol. Wt.: 1633.3949

Compound ***(R)*-2** was obtained as a dark red solid (81 mg, 45 %). ^{1}H-NMR ($CDCl_3$, 300 MHz, 323 K) δ 8.82 (2H, H_3, s), 8.76 (2H, H_1, d, J = 8.0 Hz), 8.65 (2H, H_2, d, J =8.0 Hz), 6.73 (4H, H_4, s), 4.83 (2H, H_j, t, J = 6.8 Hz), 4.17–4.03 (4H, $H_{a'}$, m), 3.97 (8H, H_a, t, J = 6.3 Hz), 2.14–2.03 (2H, H_k, m), 2.00–1.75 (8H, $H_{b'+c'+h'}$, m), 1.73–1.44 (16H, H_{b+c+h}, m), 1.43–1.08 (50H, $H_{(e-g)+(l-r)}$, m), 0.99 (6H, $H_{d'}$, d, J = 6.6 Hz), 0.94–0.87 (24H, $H_{d+i'}$, m), 0.85 (24H, H_i, d, J = 6.6 Hz), 0.8 (3H, H_s, t, J = 7.0 Hz). ^{13}C-NMR ($CDCl_3$, 75 MHz, 298K) δ 165.2, 163.9, 153.9, 138.2, 134.5, 132.6, 131.0, 127.8, 124.3, 123.8, 122.5, 121.9, 121.4, 119.1, 118.5, 107.2, 77.4, 71.9, 67.6, 39.6, 39.5, 37.8, 37.7, 37.6(1), 37.6(0), 36.6, 31.9, 30.1, 30.0, 29.5, 29.4, 29.3, 29.2, 28.2, 28.1, 27.2, 24.9, 24.8, 22.9, 22.8(6), 22.8(2), 22.7(5), 22.7(1), 19.8, 19.7, 14.2. FTIR (neat) 2954, 2925, 2869, 1699, 1663, 1600, 1559, 1503, 1466, 1438, 1424, 1398, 1380, 1366, 1303, 1233, 1174, 1172, 1112, 1048, 999, 968, 902, 843, 804, 740, 711, 685 cm^{-1}. HRSM (MALDI-TOF, exact mass) calcd. for $C_{106}H_{157}N_3O_{10}$ $[M+H]^+$, 1633.1869; found, 1633.1947.

3. Collection of spectra

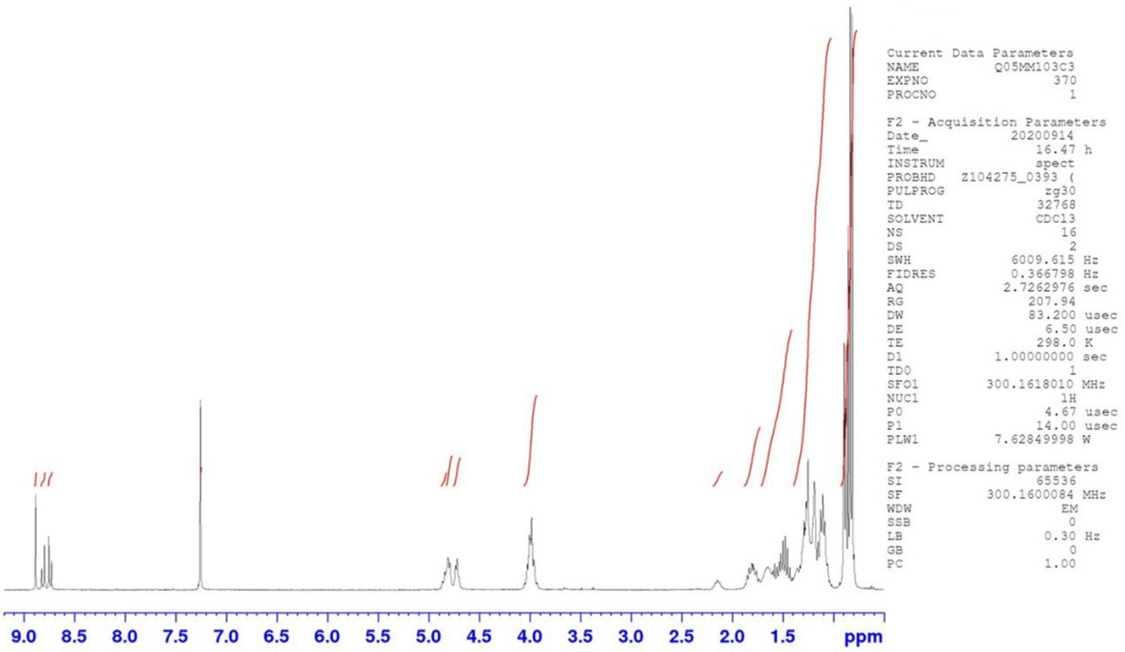

^{1}H NMR ($CDCl_3$, 300 MHz, 298 K) of compound ***(S)*-1**

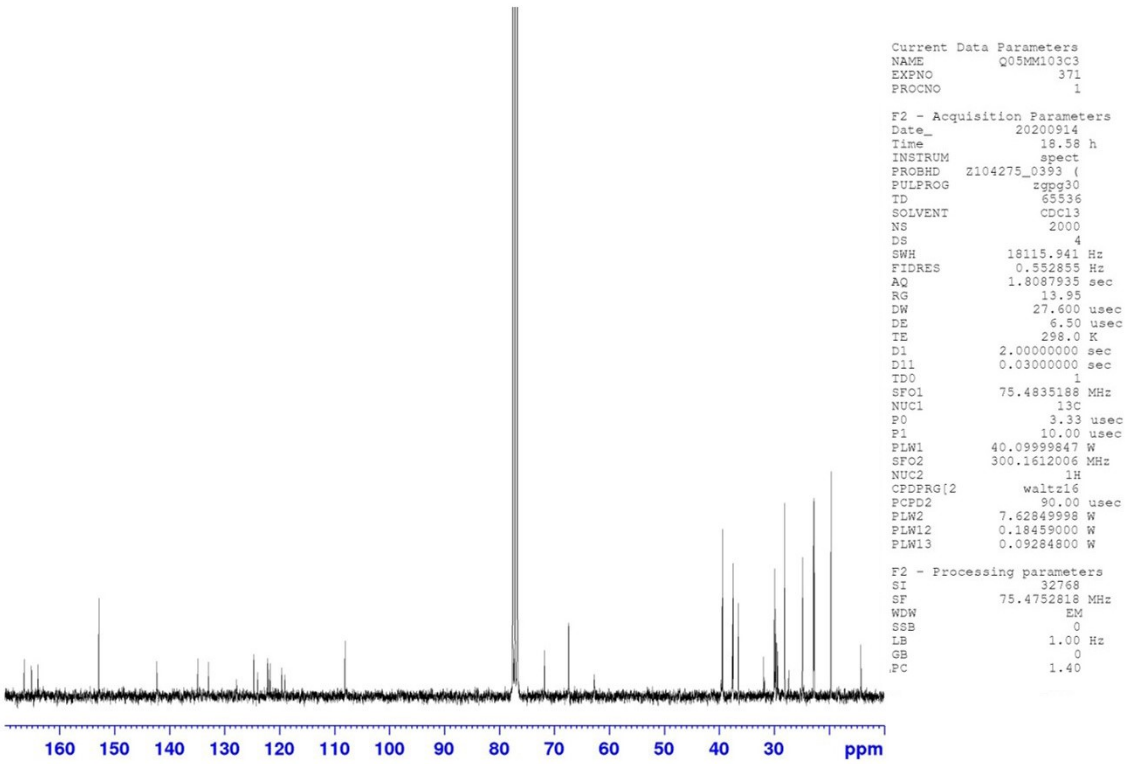

^{13}C NMR ($CDCl_3$, 75 MHz, 298 K) of compound ***(S)*-1**

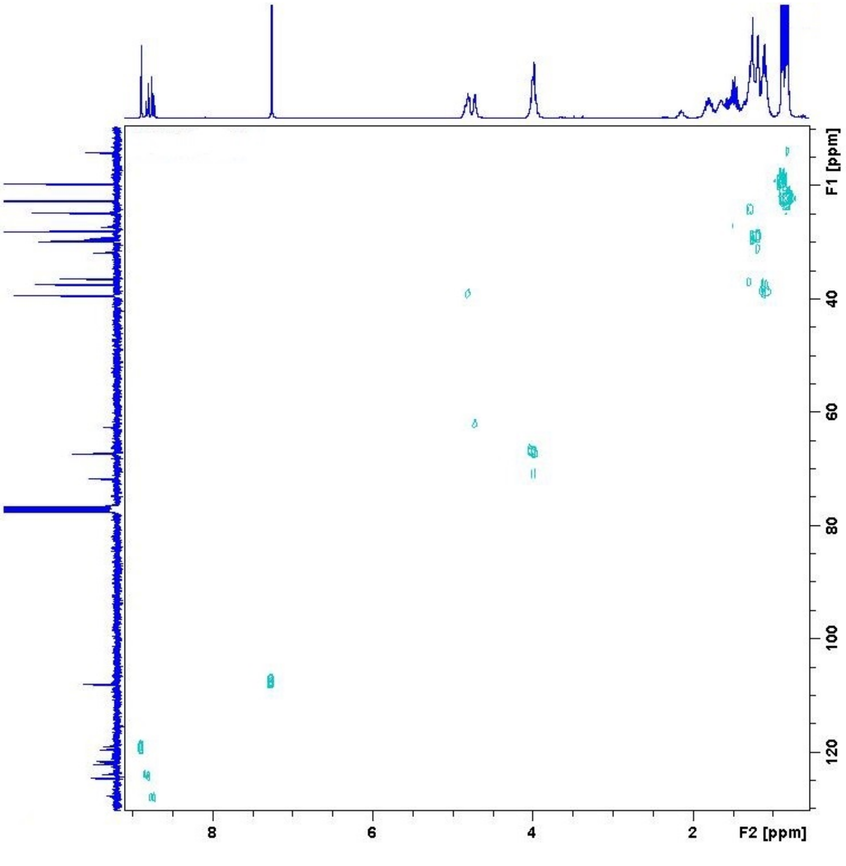

^{1}H,^{13}C-HMQC spectrum ($CDCl_3$, 298 K) of compound ***(S)*-1**

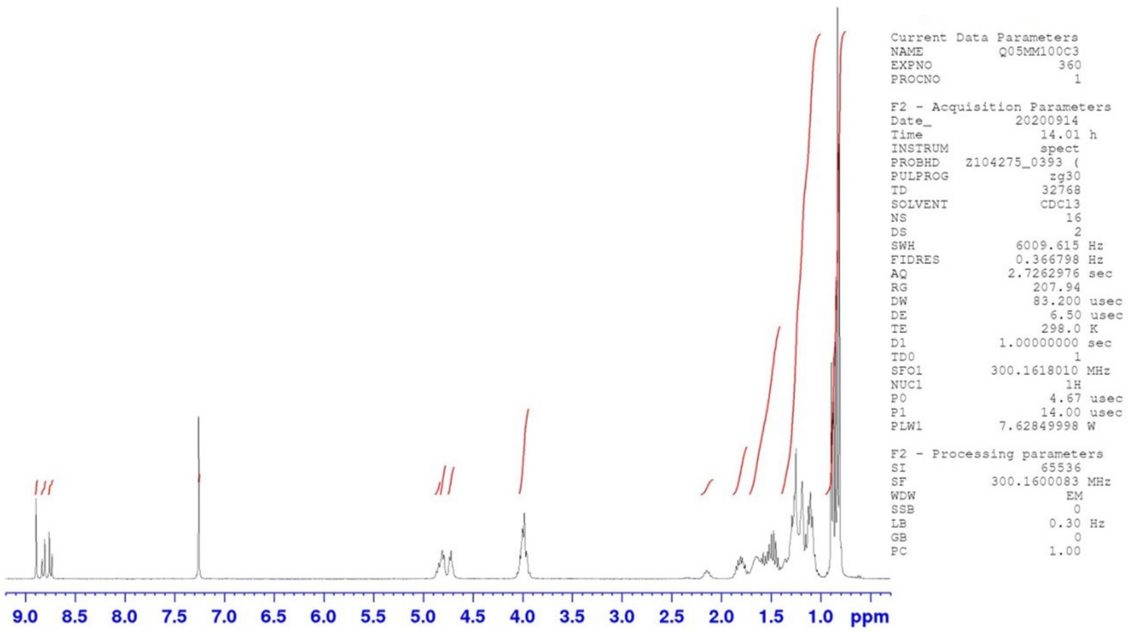

^{1}H NMR ($CDCl_3$, 300 MHz, 298 K) of compound ***(R)*-1**

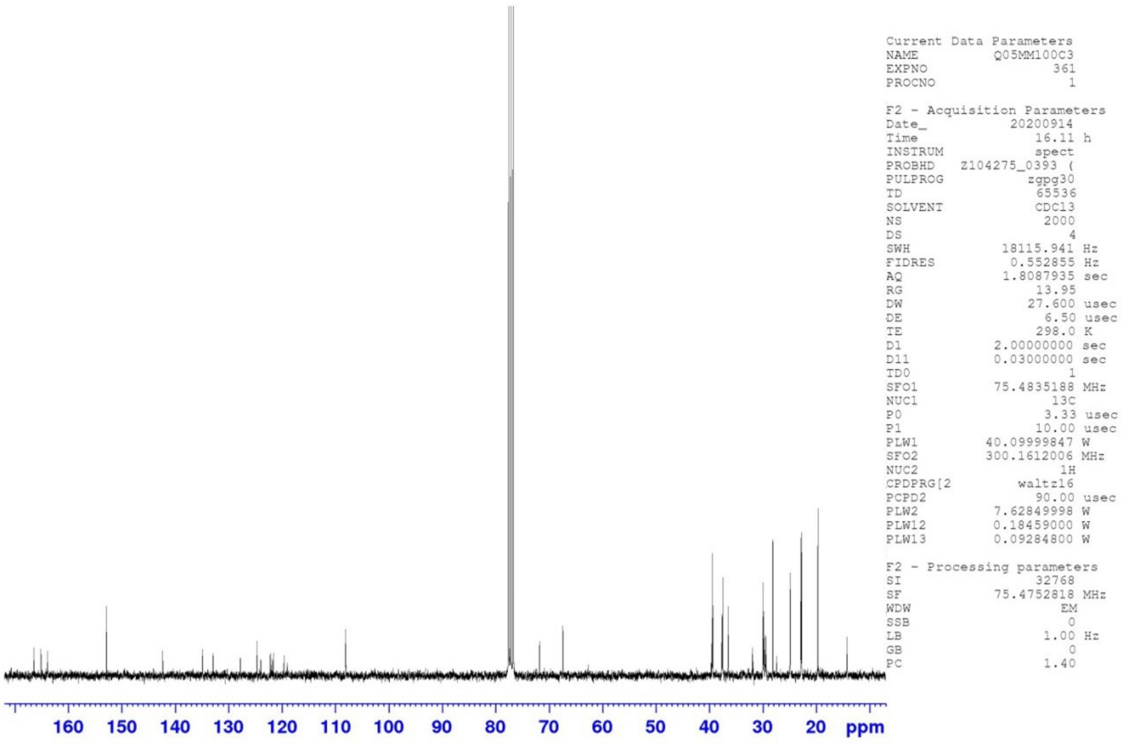

^{13}C NMR ($CDCl_3$, 75 MHz, 298 K) of compound ***(R)*-1**

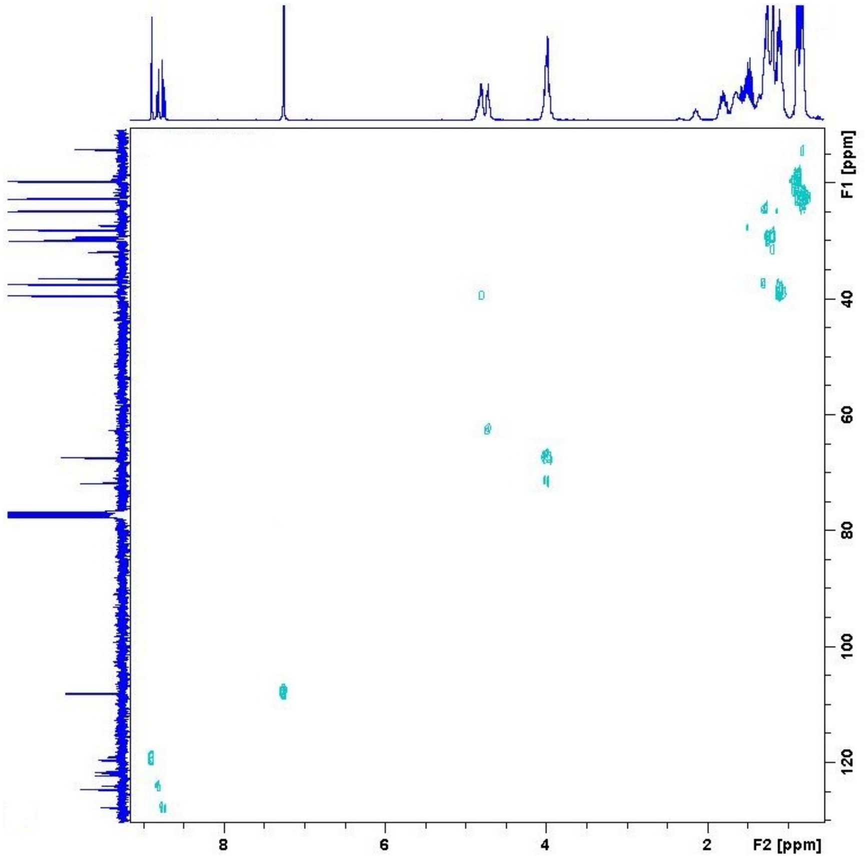

$^1H,^{13}C$-HMQC spectrum ($CDCl_3$, 298 K) of compound ***(R)*-1**

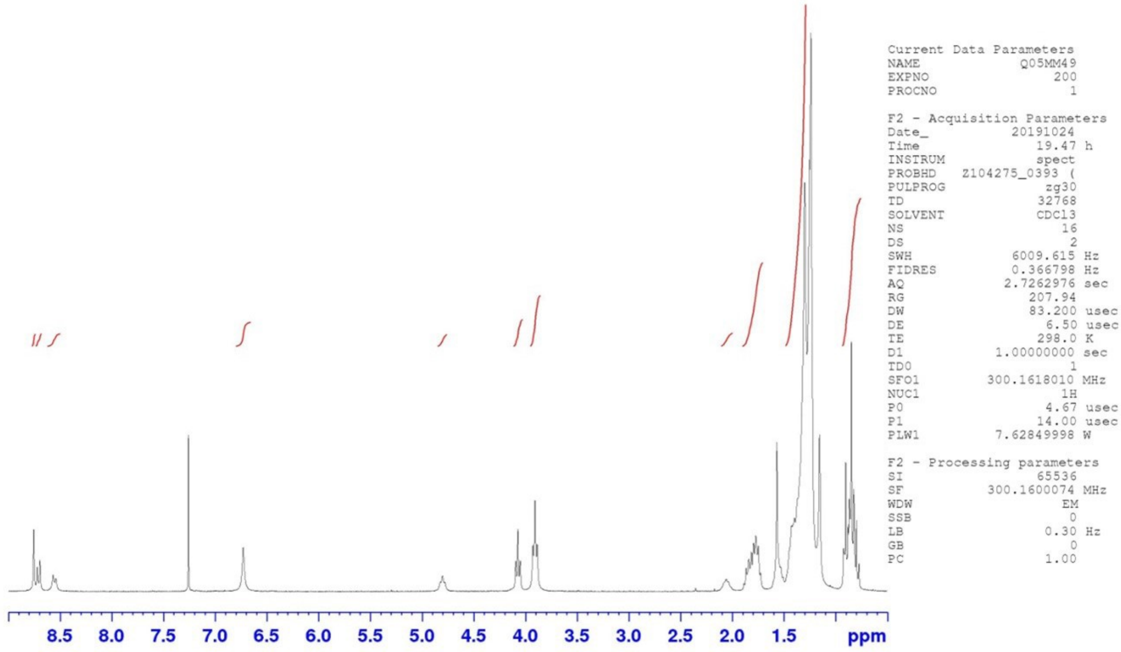

^1H NMR ($CDCl_3$, 300 MHz, 298 K) of compound **2**

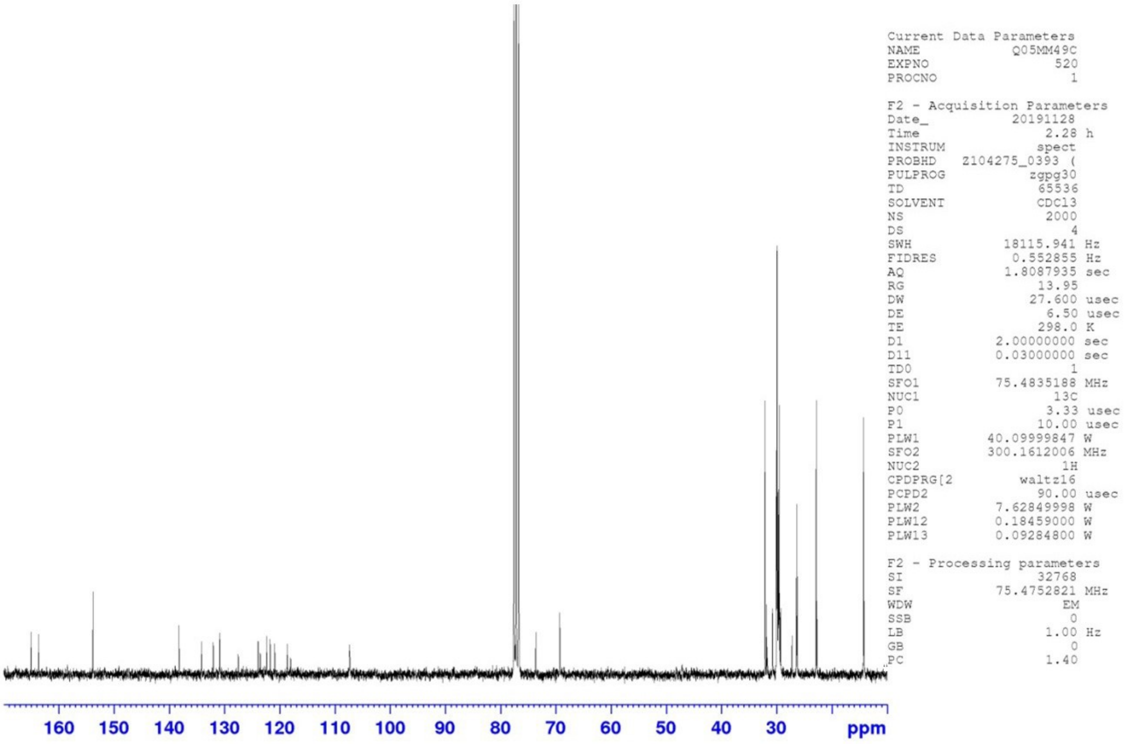

^{13}C NMR ($CDCl_3$, 75 MHz, 298 K) of compound **2**

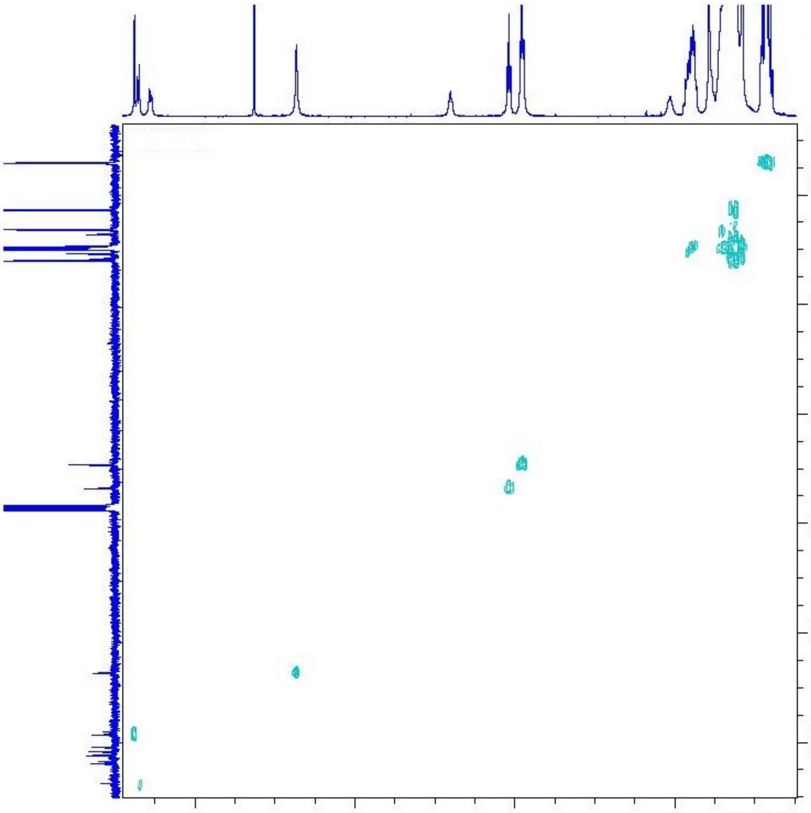

^{1}H,^{13}C-HMQCspectrum ($CDCl_3$, 298 K) of compound **2**

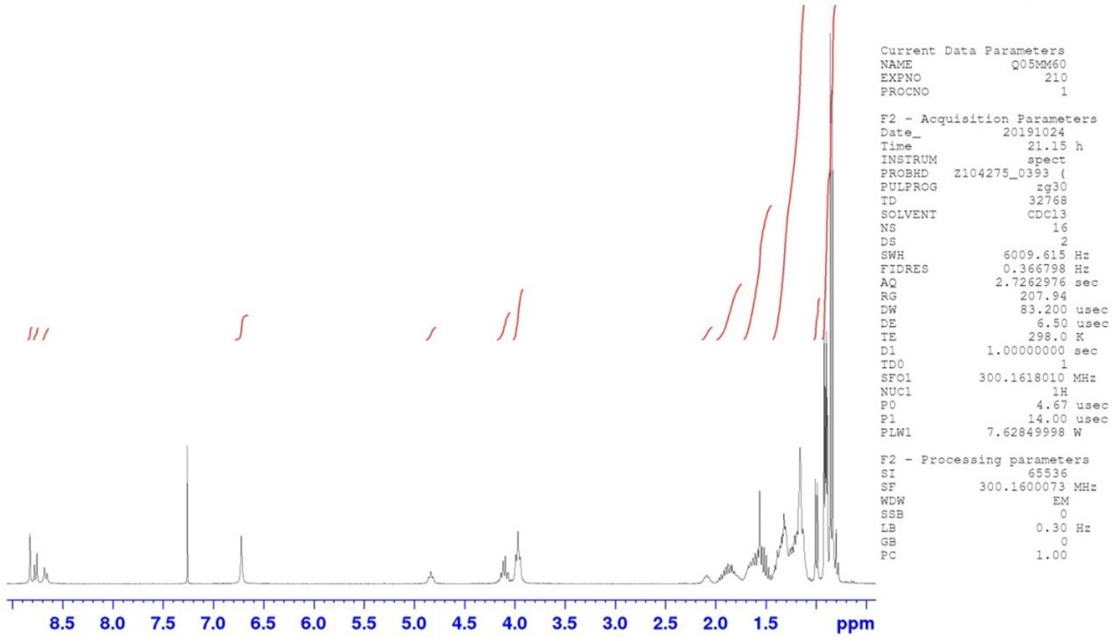

^{1}H NMR ($CDCl_3$, 300 MHz, 298 K) of compound ***(S)*-2**

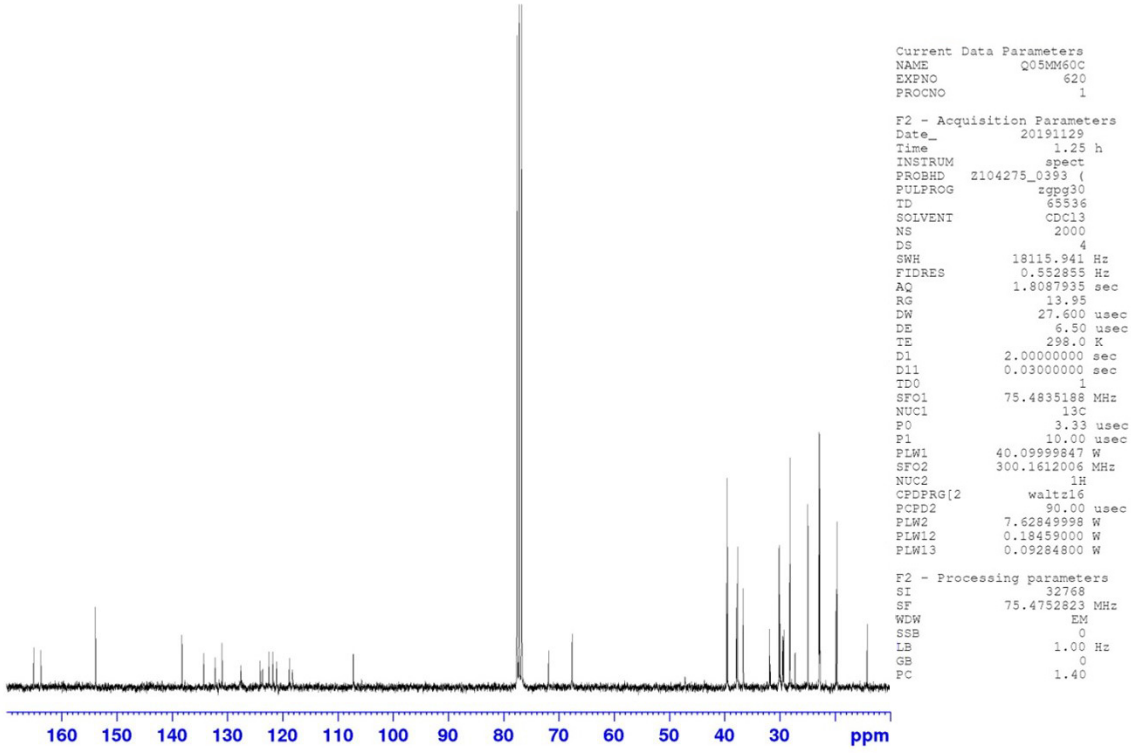

^{13}C NMR ($CDCl_3$, 75 MHz, 298 K) of compound ***(S)*-2**

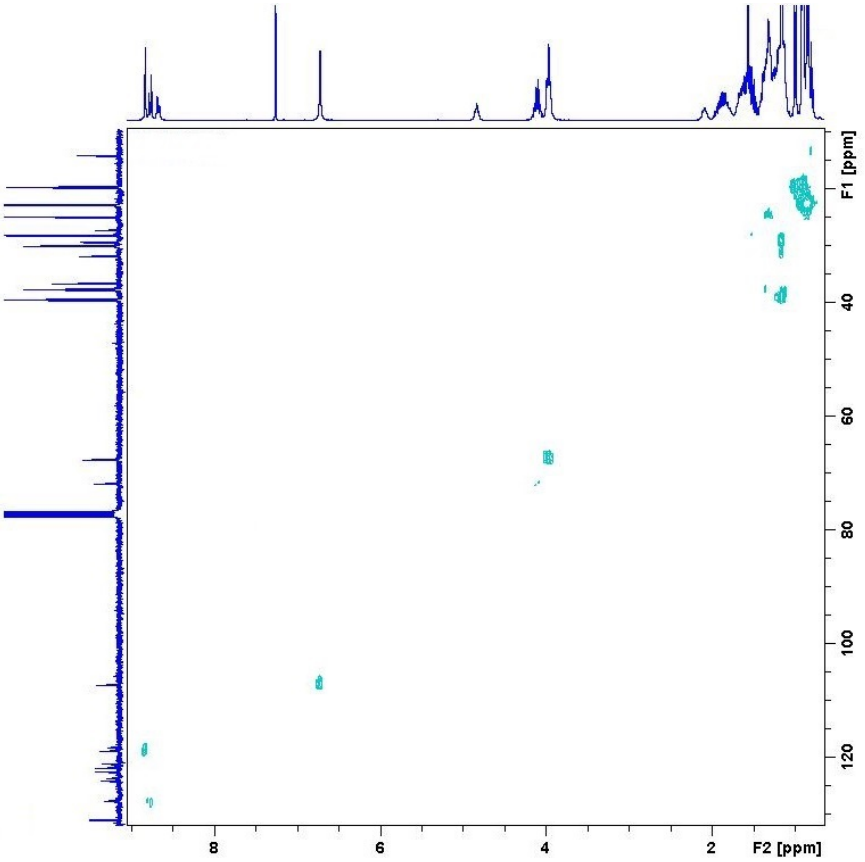

$^{1}H,^{13}C$-HMQCspectrum ($CDCl_3$, 298 K) of compound ***(S)*-2**

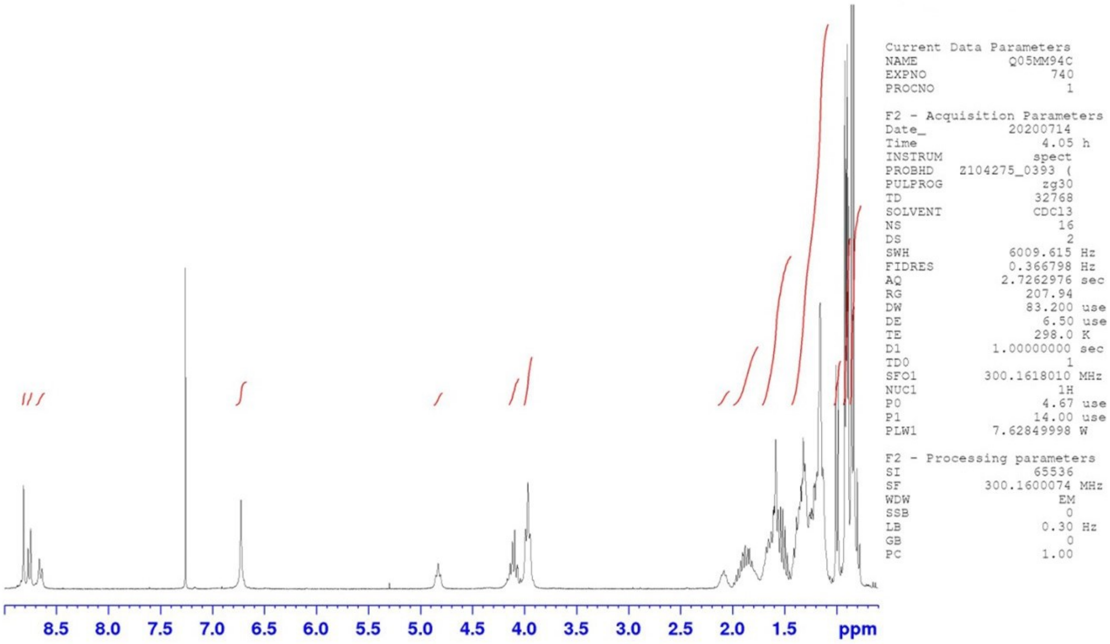

^{1}H NMR ($CDCl_3$, 300 MHz, 298 K) of compound ***(R)*-2**

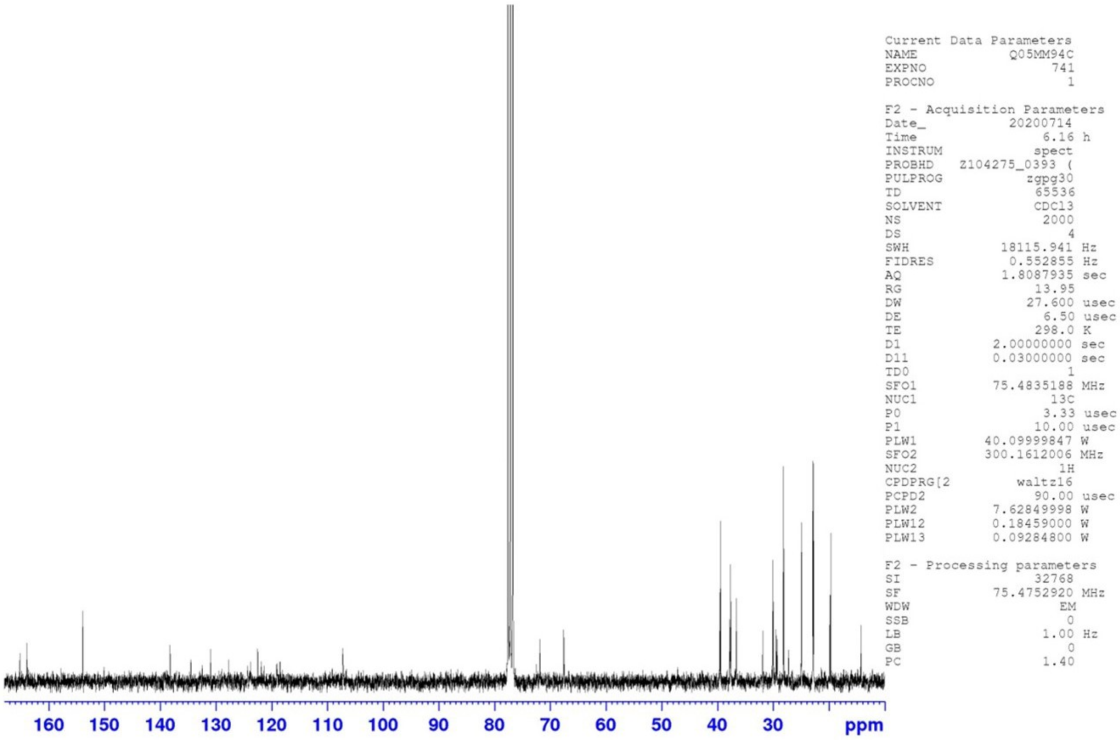

^{13}C NMR ($CDCl_3$, 75 MHz, 298 K) of compound ***(R)*-2**

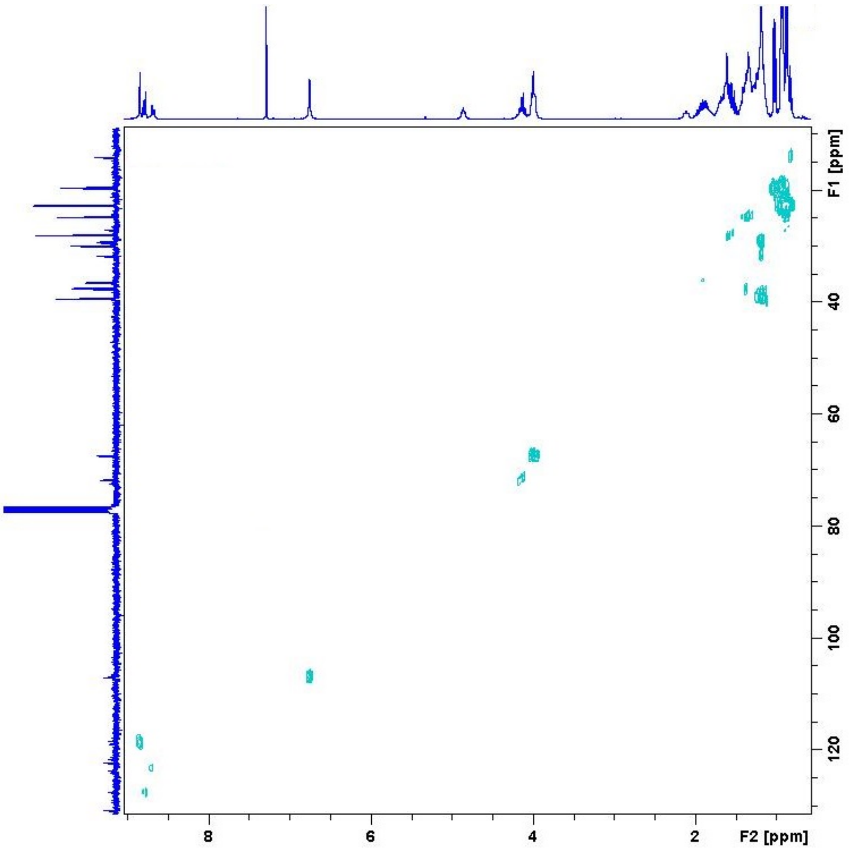

^{1}H,^{13}C-HMQCspectrum ($CDCl_3$, 298 K) of compound ***(R)*-2**

4. Supplementary Figures and Tables

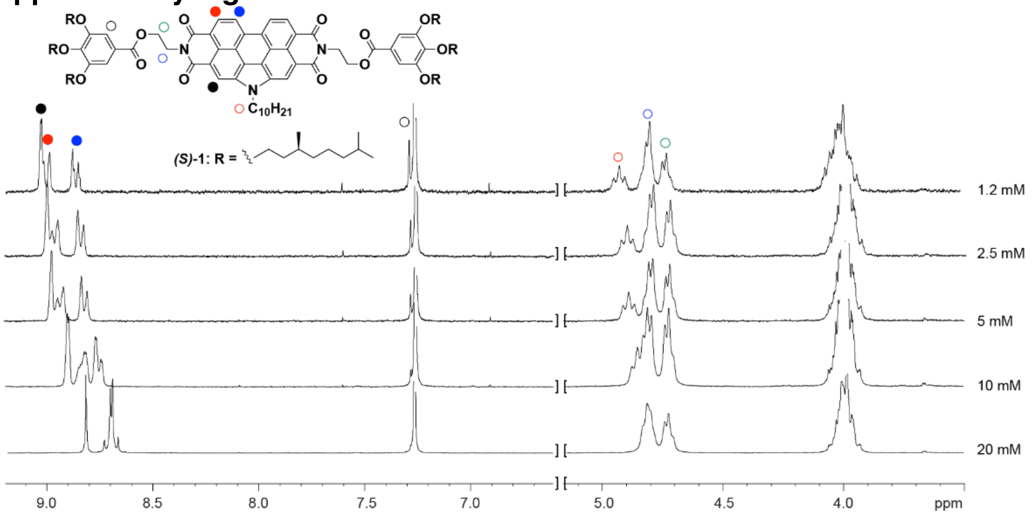

Figure S1. Partial ^{1}H NMR spectra of ***(S)*-1** recorded at different concentrations showing the aromatic and some of the aliphatic protons ($CDCl_3$, 300 MHz, 298 K).

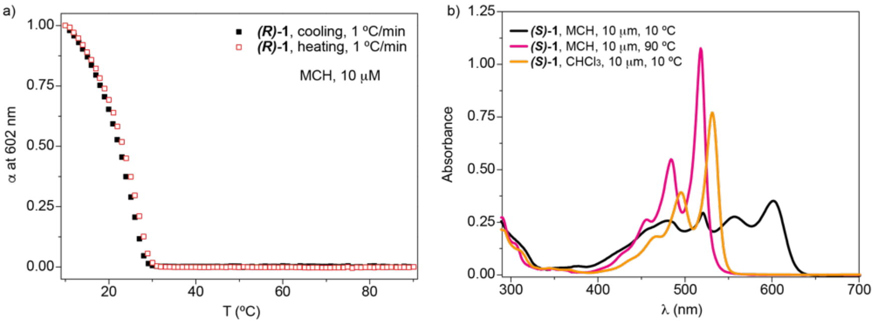

Figure S2. (a) Cooling and heating curves obtained by plotting the variation of the degree of polymerization (α) at 602 nm versus temperature for ***(R)*-1** in MCH (cooling and heating at 1 ºC min^{-1}). (b) UV-Vis spectra of ***(S)*-1** in $CHCl_3$ and MCH at c_T = 10 μM.

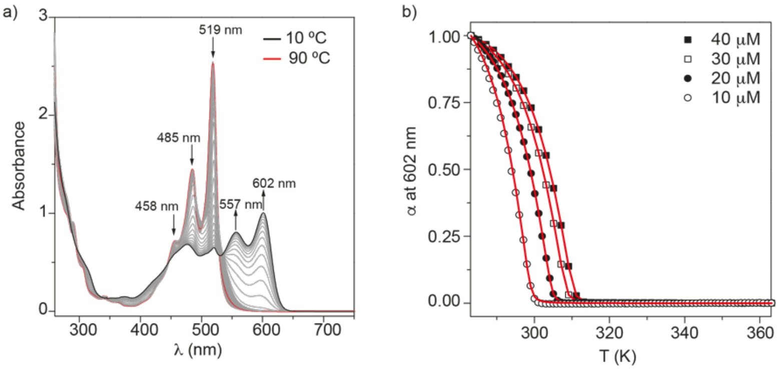

Figure S3. a) UV-Vis spectra of ***(R)*-1** at different temperatures (MCH, c_T = 10 μM). b) Plot of the variation of the degree of polymerization (α) versus temperature cooling at 1 ºC min^{-1}. Red curves correspond to the fitting to the EQ model.

Table S1. Photophysical Parameters for compounds ***(S)*-1** and ***(S)*-2** in their monomeric and aggregated states

Compound	Concentration (μM)	Solvent	Φ_{lum}	τ (ns)
***(S)*-1**	10	$CHCl_3$	0.191	1.91
***(S)*-1**	10	MCH	0.716	5.7
***(S)*-2**	100	$CHCl_3$	0.017	1.1
***(S)*-2**	100	MCH	0.002	1.3
***(S)*-2**	500	Tol	0.004	1.3

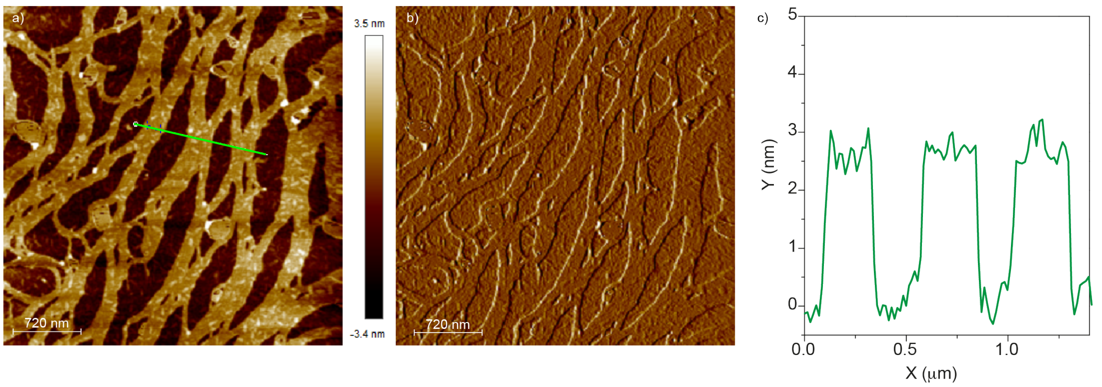

Figure S4. Height (a) and phase (b) AFM images of the bundles of fibers formed by ***(S)*-1**. (c) Height profile of the bundles along the green line in (a). Experimental conditions: HOPG as surface, MCH, c_T = 10 μm.

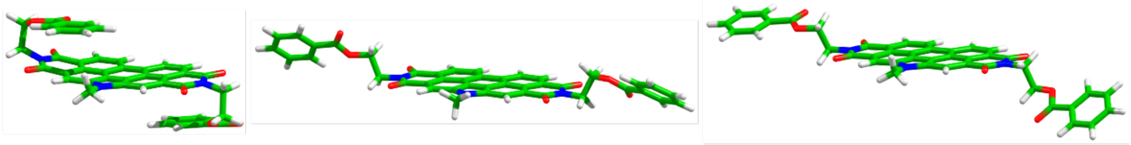

Figure S5. Minimum-energy structures (with their relative energy indicated) computed in gas phase at the GFN2-xTB level for the most stable conformers of monomer **1**.

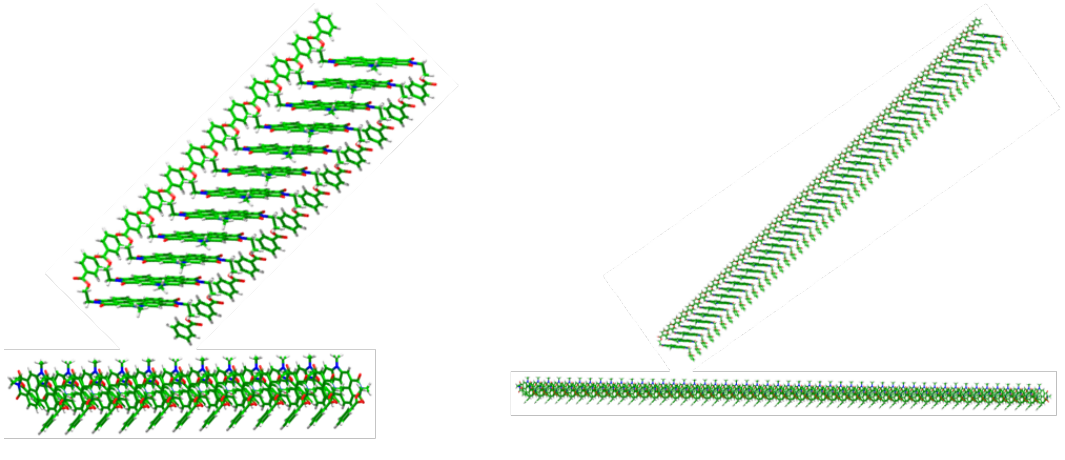

Figure S6. Optimized geometries for a 12-mer and a 50-mer J-type aggregate of compound **1** showing the staircase-like helical arrangement.

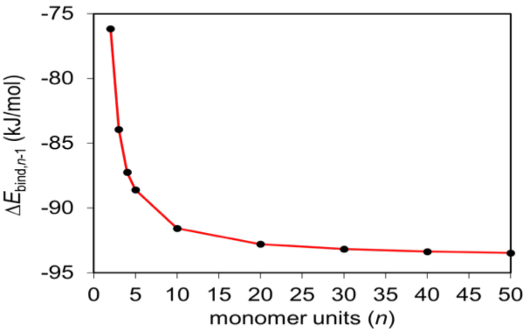

Figure S7. Evolution of the binding energy per interacting pair ($\Delta E_{bind,n\text{-}1}$) calculated at the GFN2-xTB level in n-hexane for **1A*n*** aggregates upon increasing the number of monomers (*n*) constituting the supramolecular oligomer.

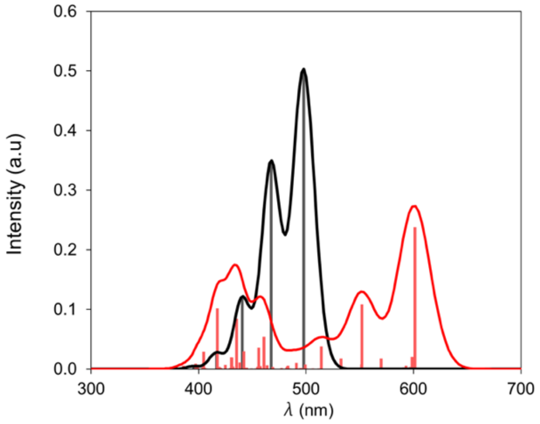

Figure S8. Simulated absorption spectrum calculated for an ideal **1A$_{10}$** oligomer by using the vibronic excitonic model explained in Section 5. The absorption spectrum computed for a monomeric unit of the *N*-annulated PBI core is included for comparison purposes.

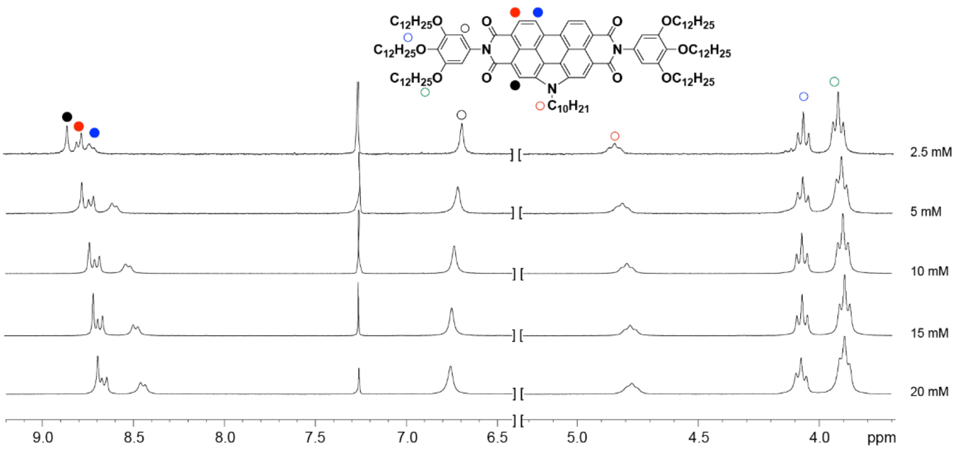

Figure S9. Partial ^{1}H NMR spectra of **2** registered at different concentrations showing the aromatic and some of the aliphatic protons ($CDCl_3$, 300 MHz, 298 K).

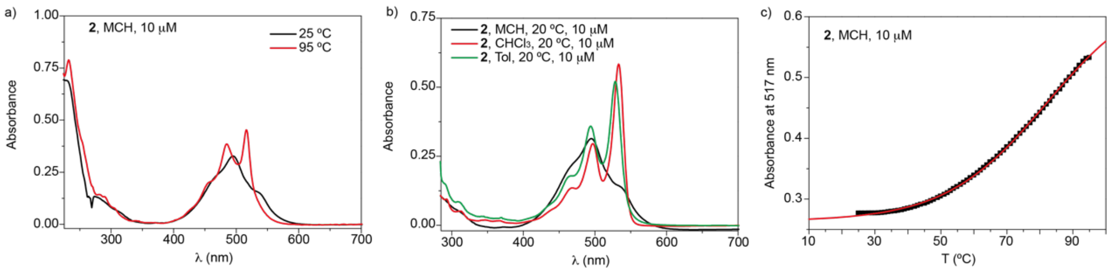

Figure S10. UV-Vis spectra of **2** at different temperatures in MCH (a) and at 20 °C in different solvents (b). (c) Plot of the variation of the absorbance measured at 517 nm versus temperature cooling at 1 °C min^{-1}. The red curve corresponds to a sigmoidal fitting to guide the eye.

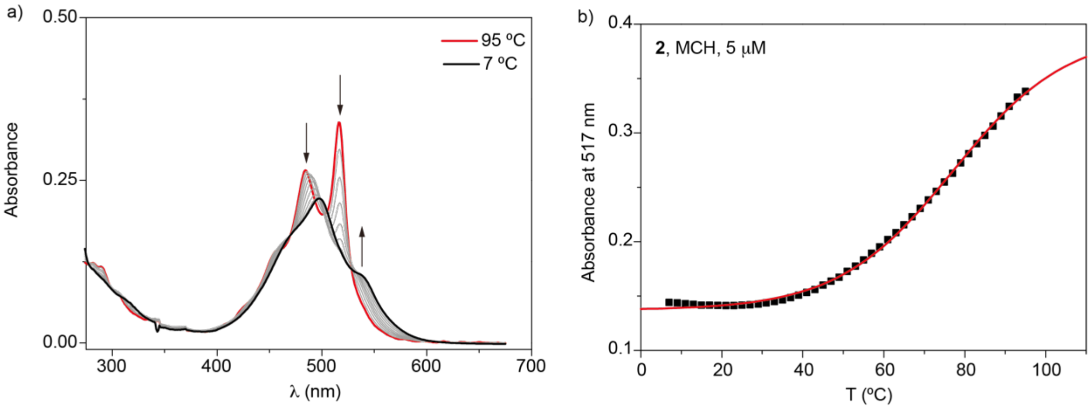

Figure S11. (a) UV-Vis spectra of **2** at different temperatures in MCH at $c_T = 5$ µM. Arrows indicate the changes in the absorption spectra upon decreasing the temperature. (b) Plot of the variation of the absorbance measured at 517 nm versus temperature cooling at 1 °C min^{-1}. The red curve corresponds to a sigmoidal fitting to guide the eye.

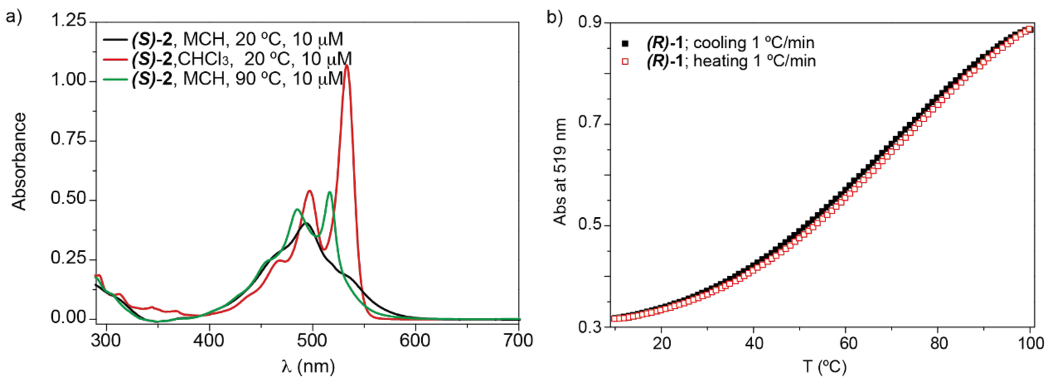

Figure S12. (a) UV-Vis spectra of ***(S)*-2** in MCH and $CHCl_3$ at c_T = 10 μM. (b) Cooling and heating curves obtained by plotting the variation of the absorbance at 519 nm versus temperature for ***(S)*-2** in decaline (cooling and heating at 1 ºC min^{-1})

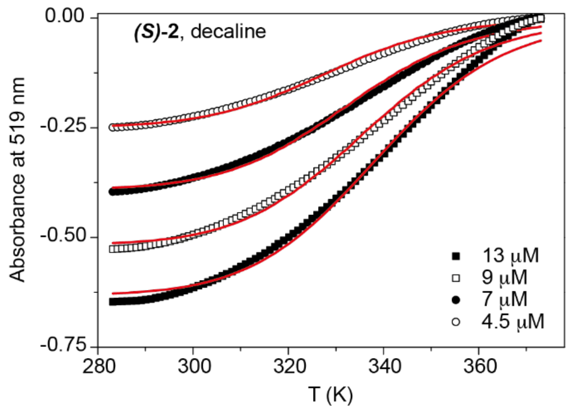

Figure S13. Plot of the variation of the absorbance measured at 519 nm for ***(S)*-2** in decaline versus temperature cooling at 1 ºK min^{-1}. Red curves correspond to the fitting to the EQ model.

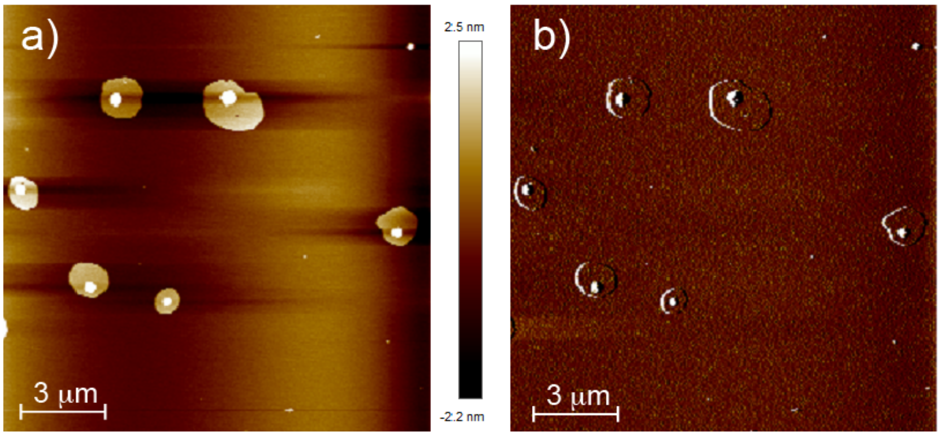

Figure S14. Height (a) and phase (b) AFM images of the aggregates formed by ***(R)*-2**. Experimental conditions: HOPG as surface, MCH, c_T = 10 μm.

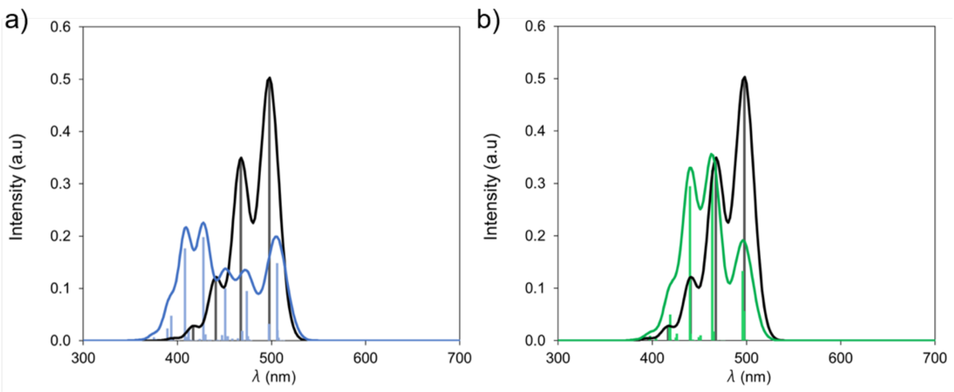

Figure S15. Simulated absorption spectra calculated for ideal **$2A_{10}$** (a) and **$2B_{10}$** (b) oligomers by using the vibronic excitonic model explained in Section 5. The absorption spectrum computed for a monomeric unit of the *N*-annulated PBI core is included for comparison purposes.

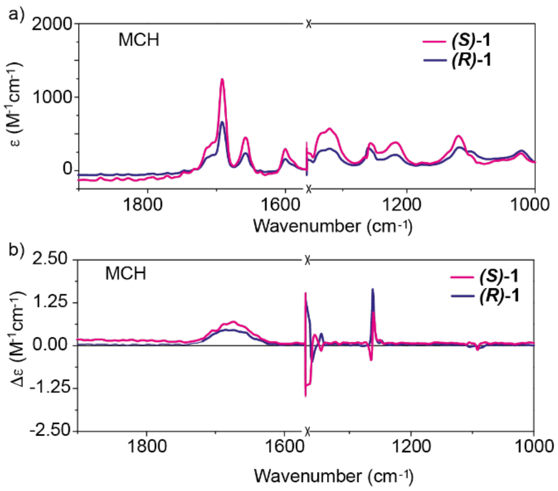

Figure S16. FTIR (a) and VCD (b) spectra of ***(S)*-1** and ***(R)*-1** in MCH at $c_T = 0.01$ M.

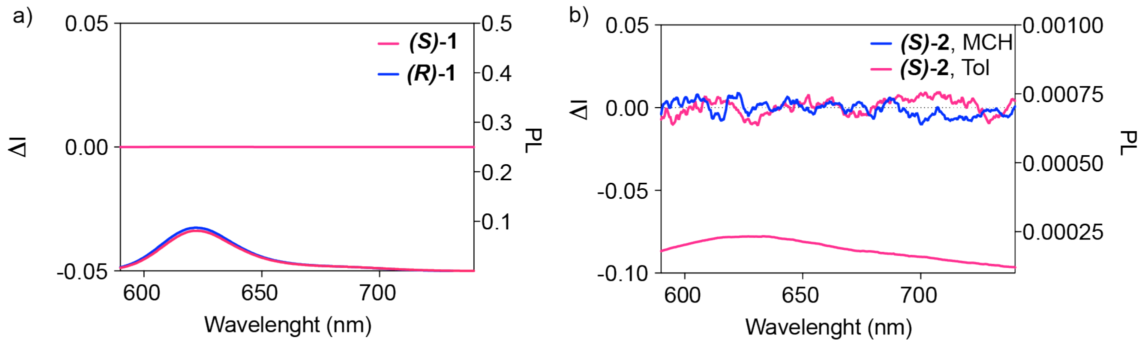

Figure S17. Emission and CPL spectra of ***(S)*-1** and ***(R)*-1** in MCH at $c_T = 10$ μM (a) and of ***(S)*-2** in MCH and Tol at $c_T = 10$ μM.

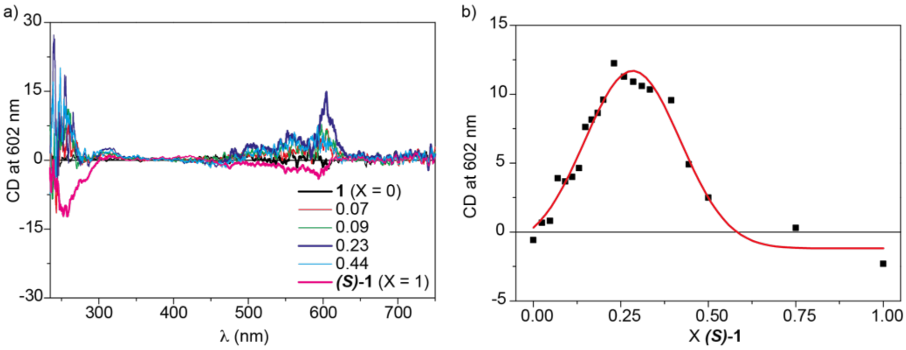

Figure S18. Selected ECD spectra registered for the SaS experiments performed by mixing **1** and ***(S)*-1** (a) and changes in CD intensity at 602 nm as a function of the molar fraction (X) of the chiral sergeant ***(S)*-1**. The red line in (b) corresponds to a gaussian fitting to guide the eye. Experimental conditions: MCH; c_T = 50 μM, 20 °C.

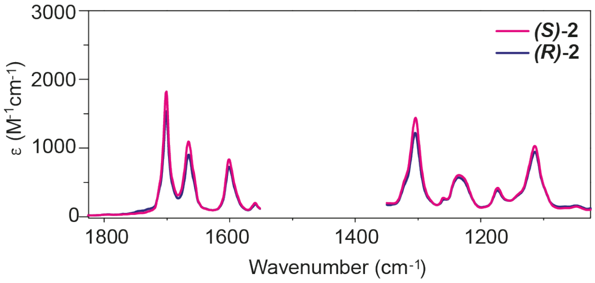

Figure S19. FTIR spectra of ***(S)*-2** and ***(R)*-2** in MCH at c_T = 0.02-0.03 M.

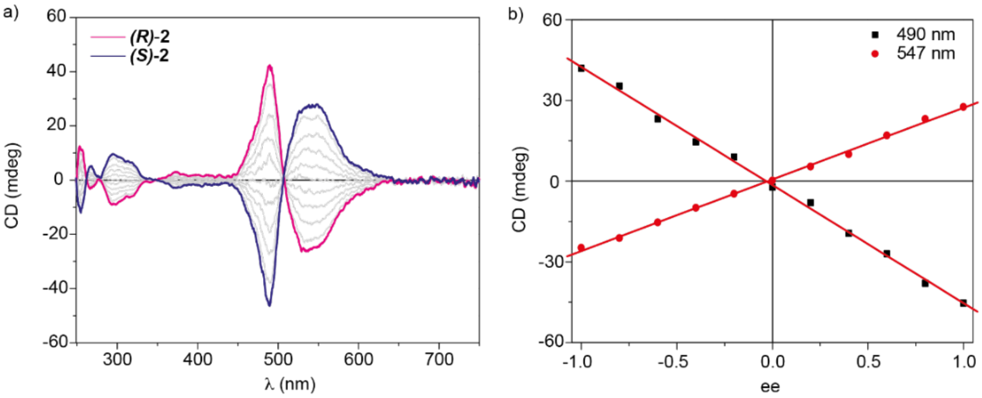

Figure S20. ECD spectra (a) and changes in ECD intensity as a function of the enantiomeric excess (*ee*) (b) upon adding ***(R)*-2** to a solution of ***(S)*-2** (MCH, c_T = 50 μM, 20 °C). *ee* = 1.0 corresponds to pure ***(R)*-2** and ee = –1.0 corresponds to pure ***(S)*-2**. Red lines correspond to linear fittings to guide the eye.

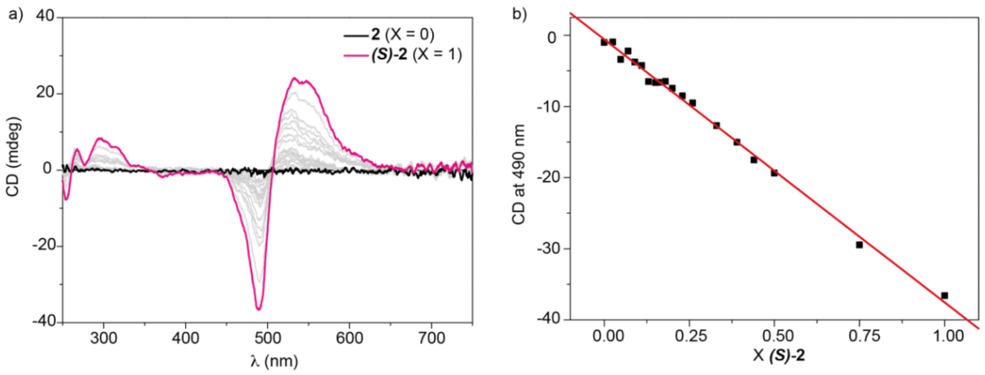

Figure S21. (a) ECD spectra registered for the SaS experiments performed by mixing **2** and ***(S)*-2**. (b) Changes in ECD intensity at 490 nm as a function of the molar fraction (*X*) of the chiral sergeant ***(S)*-2**. The red line in (b) corresponds to a linear fitting to guide the eye. Experimental conditions: MCH; c_T = 50 μM, 20 °C.

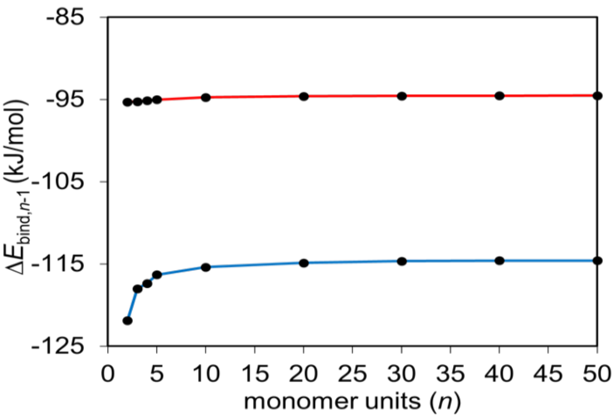

Figure S22. Evolution of the binding energy per interacting pair ($\Delta E_{\mathrm{bind},n\text{-}1}$) calculated at the GFN2-xTB level for **2A*n*** aggregates in toluene (red line) and **2B*n*** aggregates in *n*-hexane (blue line) upon increasing the number of monomers (*n*) constituting the supramolecular oligomer.

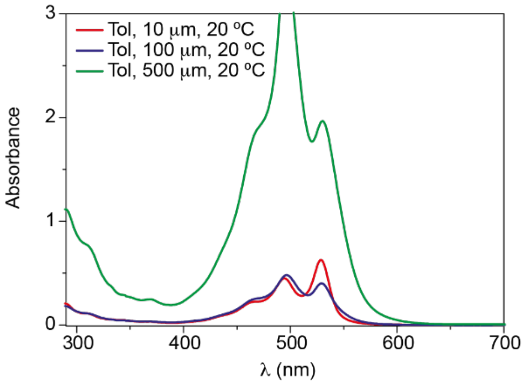

Figure S23. UV-Vis spectra of ***(S)*-2** in Tol at different concentrations.

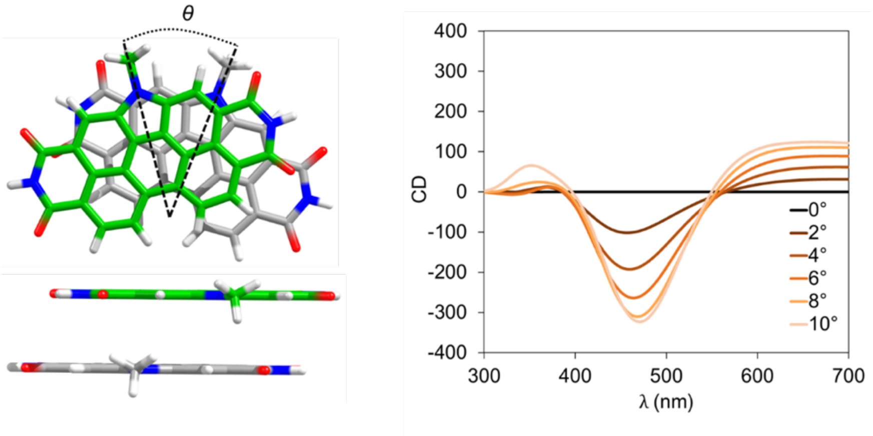

Figure S24. Simulated ECD spectra calculated at the B3LYP/6-31G** level for a dimer of the *N*-annulated PBI core at different rotational *θ* angle values.

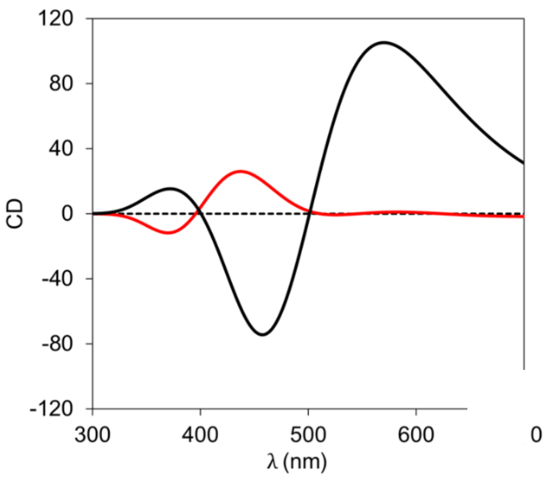

Figure S25. Simulated ECD spectra calculated at the B3LYP/6-31G** level for trimers of aggregates **2A**$_5$ (red) and **2B**$_5$ (black) optimized at the B3LYP-D3/6-31G** level including the dispersion D3 term.

5. Theoretical Calculations

The conformational space of a simplified monomeric unit of **1**, where the long alkoxy chains attached to the peripheral benzene rings are removed and the aliphatic $C_{10}H_{21}$ chain of the pyrrolic central unit is substituted by a methyl group, was explored through the Conformer–Rotamer Ensemble Sampling Tool (CREST) utility using the xtb-6.3.3 program package.[S5] Figure S5 displays the most stable conformers found for **1** after geometry optimization in gas phase at the semiempirical GFN2-xTB level of theory as implemented in the xtb program.[S6] The GFN2-xTB method is based on a Hamiltonian similar to the well-known DFTB3,[S7] with a minimal valence basis set centered on atoms (STO-mG), and includes the density-dependent D4 dispersion correction. Among the different conformers, only conformers II and III are able to self-assemble in a supramolecular polymer and their relative energies decrease to 27.26 (II) and 27.83 kJ mol^{-1} (III) when calculated in *n*-hexane as solvent. Thus, structures II and III were used to model pentamers of **1**, growing either linearly or in helical arrangements. However, attention is only paid to aggregates derived from conformer III, with the peripheral phenyl rings pointing in opposite directions with respect to the PBI plane, because those obtained from conformer II are significantly higher in energy. For instance, the pentamer of conformer II analogous to **1A$_5$** in Figure 3a is 140.94 kJ mol^{-1} higher in energy. Similarly, the minimum-energy structure of **2** was calculated at the GFN2-xTB quantum-chemical method without the alkyl chains. The two possible conformers, with the peripheral benzene rings oriented in an eclipsed or staggered disposition with respect to the long axis of the *N*-annulated PBI core axis, were computed practically isoenergetic and were further used to model supramolecular pentamers that grow up linearly or in an helical disposition, respectively (Figure 5a). The minimum-energy geometry of each stack was obtained after full geometry relaxation in gas phase at the GFN2-xTB level. The most stable supramolecular aggregates of each type, long-axis displaced pentamer **1A$_5$** (Figure 3a), helical **1B$_5$** (Figure 3b), short-axis displaced **2A$_5$** (Figure 5b), and helical **2B$_5$** (Figure 5c) were further analyzed. The gas-phase-optimized pentamers of **1** and **2** were used to perform geometry optimizations at the same level (GFN2-xTB) including solvent effects by means of the generalized Born surface area (GBSA) formulation, with *n*-hexane (parametrized solvent similar in nature to methylcyclohexane) and toluene as solvents. Frequency calculations were additionally carried out to obtain the Gibbs free energy of the aggregation process. The binding free energy per interacting pair ($\Delta G_{\mathrm{bind},n-1}$) in *n*-hexane and toluene was calculated according to

$$\Delta G_{\text{bind},n-1} = \frac{G_{\text{stack}} - n \cdot G_{\text{monomer}}}{n-1} \tag{1}$$

where G_{stack} corresponds to the free energy of the stacked aggregate with n monomeric units and G_{monomer} to the free energy calculated for the monomer. To shed light on the supramolecular mechanism of the self-assembling process of compounds **1** and **2**, interaction energy calculations were performed for regular oligomers of increasing size (from n = 1 to 50 monomers) at the GFN2-xTB level in gas phase. The intermolecular geometry parameters used for building up the ideal regular oligomers were extracted from the central part of fully optimized oligomers of 50 units. The binding energy per interacting pair ($\Delta E_{\text{bind},n\text{-}1}$) was calculated in a similar way to $\Delta G_{\text{bind},n-1}$ as

$$\Delta E_{\text{bind},n-1} = \frac{E_{\text{stack}} - n \cdot E_{\text{monomer}}}{n-1} \tag{2}$$

where E_{stack} is the total energy of the stacked aggregate with n monomeric units and E_{monomer} is the energy calculated for the monomer.

To understand the changes observed experimentally in the optical properties associated to the supramolecular polymers formed by compounds **1** and **2**, a vibronic Hamiltonian similar in spirit to that proposed by F. Spano and co-workers was used to calculate the UV-Vis spectra of the aggregates.[S8] The vibronic Hamiltonian can be decomposed as follows:

$$\hat{H} = \hat{H}_{\text{FE}} + \hat{H}_{\text{CT}} + \hat{H}_{\text{FE-CT}}\,. \tag{3}$$

In Eq. 3, $\hat{H}_{\text{FE}}$ is the usual Frenkel exciton Hamiltonian

$$\hat{H}_{\text{FE}} = \sum_{i,\tilde{v}} \left(E_i - \Delta + \tilde{v}\hbar\omega_{\text{eff}}\right) |i,\tilde{v}\rangle\langle i,\tilde{v}| + \sum_{i,j\neq i}\sum_{\tilde{v},\tilde{w}} J_{ij} \langle\tilde{v}|0\rangle\langle 0|\tilde{w}\rangle |i,\tilde{v}\rangle\langle j,\tilde{w}| \tag{4}$$

where $|i,\tilde{v}\rangle$ denotes a vibronic state in which monomer i is in its first electronic excited state (in its vibrational level $\tilde{v}$) and the other monomers are in their electronic and vibrational ground states. E_i is the excitation energy localized on molecule i, where the zero-point energy has been included, and Δ is a solution-to-aggregate energy shift which accounts for nonresonant dispersion interactions between chromophores in the supramolecular assembly. $\hbar\omega_{\text{eff}}$ is the frequency of the effective intramolecular vibration (one per monomer). J_{ij} corresponds to the excitonic coupling between electronic states i and j, which is weighted by the Franck–Condon integrals (FCIs) $\langle\tilde{v}|0\rangle$ and $\langle 0|\tilde{w}\rangle$. FCIs within the displaced harmonic oscillator model depends on the Huang–Rhys (HR) factors

between the involved electronic states.[S9] In our case, the HR factor between the ground state and the first electronic state S_{HR}^{*} was used.

$\hat{H}_{\text{CT}}$ denotes the diagonal Hamiltonian involving charge transfer (CT) excited states and can be written as:

$$\hat{H}_{\text{CT}} = \sum_{i,v^+,v^-} \left(E_{CT} + \left(v^+ + v^-\right)\hbar\omega_{\text{eff}}^{\text{CT}}\right)\left(\left|i^+,v^+;(i+1)^-,v^-\right\rangle\left\langle i^+,v^+;(i+1)^-,v^-\right| + \left|i^-,v^-;(i+1)^+,v^+\right\rangle\left\langle i^-,v^-;(i+1)^+,v^+\right|\right) \quad (5)$$

where $\left|i^+,v^+;(i+1)^-,v^-\right\rangle$ is a two-particle charge-transfer state with a cation (anion) located in the i (i+1) molecule. E_{CT} is the energy of the CT excited states between vicinal monomers, where the cation (anion) is localized on monomer i and the anion (cation) on i+1. $\hbar\omega_{\text{eff}}^{\text{CT}}$ is the frequency of an effective vibration for the cation and anion states, which are assumed to be equivalent. v^+ and v^- correspond to the vibrational levels of the cation and anion states, respectively.

Finally, the $\hat{H}_{\text{FE-CT}}$ term denotes the Frenkel/CT coupling Hamiltonian, which can be written as follows:

$$\begin{aligned} \hat{H}_{\text{FE-CT}} &= \sum_{i,\tilde{v}} \sum_{v^+,v^-} t_{\text{e}} \left\langle \tilde{v} \middle| v^+ \right\rangle \left\langle 0 \middle| v^- \right\rangle \left|i,\tilde{v}\right\rangle \left\langle i^+,v^+;(i+1)^-,v^-\right| + \text{h.c.} \\ &\sum_{i,\tilde{v}} \sum_{v^+,v^-} t_{\text{h}} \left\langle \tilde{v} \middle| v^- \right\rangle \left\langle 0 \middle| v^+ \right\rangle \left|i,\tilde{v}\right\rangle \left\langle i^-,v^-;(i+1)^+,v^+\right| + \text{h.c.} \end{aligned} \quad (6)$$

where t_{e} and t_{h} denotes the electron and hole transfer integrals, and the Franck–Condon integrals $\left\langle \tilde{v} \middle| v^+ \right\rangle$, $\left\langle \tilde{v} \middle| v^- \right\rangle$, $\left\langle 0 \middle| v^+ \right\rangle$ and $\left\langle 0 \middle| v^- \right\rangle$ depend on the HR factors $S_{\text{HR}}^{*/+}$, $S_{\text{HR}}^{*/-}$, S_{HR}^{+}, and S_{HR}^{-}, respectively (see Table S1 for the values used).

The eigenstates of the Hamiltonian in Eq. 3, for which cyclic boundary conditions were applied, can be described as a linear combination of the molecular excited states as:

$$\left|\psi_\alpha\right\rangle = \sum_{i,\tilde{v}} C_{i,\tilde{v}}^{\alpha} \left|i,\tilde{v}\right\rangle + \sum_{i} \sum_{v^+,v^-} C_{i^+,v^+;(i+1)^-,v^-}^{\alpha} \left|i^+,v^+;(i+1)^-,v^-\right\rangle \quad (7)$$

where the $C_{i,\tilde{v}}^{\alpha}$ and $C_{i^+,v^+;(i+1)^-,v^-}^{\alpha}$ coefficients are obtained by diagonalization of the Hamiltonian. The maximum number of vibrational levels ($\tilde{v}$, v^+, and v^-) was set to be 5 because it provides a nice convergence of the calculated absorption spectra without compromising the size of the vibronic Hamiltonian. The homogeneous absorption

spectrum $A(E)$ is derived from the sum over all eigenstates $|\psi_\alpha\rangle$ of the aggregate Hamiltonian as

$$A(E) = \sum_\alpha f_\alpha W_{LS}(E_\alpha - E) \quad (8)$$

where the oscillator strengths f_α are evaluated as $f_\alpha = (2/3)E_\alpha \left|\langle\psi_\alpha|\hat{\mu}|G\rangle\right|^2$. E_α is the energy of the eigenstate $|\psi_\alpha\rangle$, $|G\rangle$ denotes the state where all the molecules are in their ground state, and $\hat{\mu}$ corresponds to the electric dipole moment operator. Finally, $W_{LS}(E_\alpha - E)$ represents a Gaussian shape function.

Table S1. Parameters (in eV) used in the vibronic Hamiltonian employed to calculate the UV-Vis spectra of the supramolecular oligomers **$1A_{10}$**, **$2A_{10}$**, and **$2B_{10}$**.

Parameters	**$1A_{10}$**	**$2A_{10}$**	**$2B_{10}$**
E_i	2.38	2.38	2.38
Δ	0.200	0.050	0.000
$\hbar\omega_{\mathrm{eff}} = \hbar\omega_{\mathrm{eff}}^{\mathrm{CT}}$	0.161	0.161	0.161
J_{ij}	0.059	0.077	0.062
S_{HR}^{*} [a]	0.690	0.690	0.690
E_{CT}	2.58	2.58	2.58
t_{e}	0.101	−0.077	0.048
t_{h}	0.154	−0.070	0.002
$S_{\mathrm{HR}}^{+} = S_{\mathrm{HR}}^{-}$ [a]	0.440	0.440	0.440
$S_{\mathrm{HR}}^{*/+} = S_{\mathrm{HR}}^{*/-}$ [a,b]	0.028	0.028	0.028

[a] HR factors are adimensional. [b] $S_{\mathrm{HR}}^{*/+}$ is computed as $S_{\mathrm{HR}}^{*/+} = \left(\sqrt{S_{\mathrm{HR}}^{*}} - \sqrt{S_{\mathrm{HR}}^{+}}\right)^2$.

Table S1 gathers all the parameters used in the vibronic Hamiltonian. Most of these parameters were evaluated using data obtained from density functional theory (DFT) and time-dependent DFT (TD-DFT) calculations on monomer and dimer species of the *N*-annulated PBI core. DFT calculations were performed within the Gaussian 16 A.03 program package.[S10] The E_i energy of the first bright electronic $S_0 \rightarrow S_1$ transition was estimated from the optimization of the monomer at the TD-DFT level with the B3LYP functional[S11,S12] and the 6-31G** basis.[S13] The gas-phase energy was corrected to account for solvent effects (*n*-hexane) with the PCM approach.[S14] The diabatic E_{CT} energy is not easy to be accurately predicted by B3LYP calculations and was set to be 0.2 eV above the bright electronic $S_0 \rightarrow S_1$ transition according to the recent diabatic

calculations by Negri and co-workers.[S15] The effective frequency $\hbar\omega_{\mathrm{eff}}$ and the S_{HR}^{*} factor were derived from the experimental absorption spectrum of compound **1** (monomer) according to Spano and coworkers.[S8] $\hbar\omega_{\mathrm{eff}}^{\mathrm{CT}}$ was assumed to be equal to $\hbar\omega_{\mathrm{eff}}$ and the ionic HR factors (S_{HR}^{+} and S_{HR}^{-}) were computed to reproduce the relaxation energy of the ionic species ($\lambda_{\mathrm{rel}}^{+}$ and $\lambda_{\mathrm{rel}}^{-}$) at B3LYP/6-31G** according to $\lambda_{\mathrm{rel}}^{+} = S_{\mathrm{HR}}^{+}\hbar\omega_{\mathrm{eff}}^{\mathrm{CT}}$ and $\lambda_{\mathrm{rel}}^{-} = S_{\mathrm{HR}}^{-}\hbar\omega_{\mathrm{eff}}^{\mathrm{CT}}$. The intermolecular parameters (J_{ij}, t_{e}, and t_{h}) were evaluated by using the central dimers extracted from the previously GFN2-xTB-optimized pentamers (**1A$_5$**, **2A$_5$**, and **2B$_5$**). As GFN2-xTB level tends to slightly underestimate the intermolecular separation in π-stacks, the intermolecular separation between the *N*-annulated PBI cores were slightly elongated to 3.5 Å while maintaining the same orientation. On the basis of these corrected dimers, TD-DFT calculations were performed at the B3LYP/6-31G** level in *n*-hexane to estimate the intermolecular excitonic J_{ij} couplings by using the approximation developed by Curutchet and Mennucci (EET keyword in Gaussian).[S16] The hole and electron transfer integrals were computed by employing the projected method proposed by Baumeier *et al.* using the data obtained from B3LYP/6-31G** calculations.[S17] In the projection method, the dimer molecular orbitals are projected into the basis of the isolated-molecule molecular orbitals.

Circular dichroism spectra of monomers **1** and **2** and related oligomers were calculated at the B3LYP/6-31G** level by convoluting the lowest-lying singlet excited states with a Gaussian function broadening of FWHM = 0.2 eV. The spectra in Figure S23 are calculated for a dimer of the N-annulated PBI core at different values of the rotational angle *θ* along the growing axis. The spectra in Figure S24 are calculated for trimers of compound **2** optimized at the B3LYP-D3/6-31G** level following the aggregation pattern found for **2A$_5$** and **2B$_5$**.

References

(S1) ten Eikelder, H. M. M.; Markvoort, A. J.; de Greef, T. F. A.; Hilbers, P. A. J. An equilibrium model for chiral amplification in supramolecular polymers. *J. Phys. Chem. B* **2012**, *116*, 5291–5301.

(S2) (a) Kulkarni, C.; Munirathinam, R.; George, S. J. Self-Assembly of Coronene Bisimides: Mechanistic Insight and Chiral Amplification. *Chem. Eur. J.* **2013**, *19*, 11270–11278. (b) van den Hout, K. P.; Martín-Rapún, R.; Vekemans, J. A. J. M.; Meijer, E. W. Tuning the Stacking Properties of C3-Symmetrical Molecules by Modifying a Dipeptide Motif. *Chem. Eur. J.* **2007**, *13*, 8111–8123. (c) Kulkarni, C.; Korevaar, P. A.; Bejagam, K. K.; Palmans, A. R. A.; Meijer, E. W.; George, S. J. Solvent Clathrate Driven Dynamic Stereomutation of a Supramolecular Polymer with Molecular Pockets. *J. Am. Chem. Soc.* **2017**, *139*, 13867–13875.

(S3) (a) Greciano, E. E.; Matarranz, B.; Sánchez, L. Pathway Complexity Versus Hierarchical Self-Assembly in N-Annulated Perylenes: Structural Effects in Seeded Supramolecular Polymerization. *Angew. Chem. Int. Ed.* **2018**, *57*, 4697–4701. (b) Dorca, Y.; Naranjo, C.; Ghosh, G.; Gómez, R.; Fernández, G.; Sánchez, L. Unconventional Chiral Amplification in Luminescent Supramolecular Polymers Based on Trisbiphenylaminetricarboxamides. *Organic Materials* **2020**, *2*, 41–46. (c) Naranjo, C.; Dorca, Y.; Ghosh, G.; Gómez, R.; Fernández, G.; Sánchez, L. Chain-capper effect to bias the amplification of asymmetry in supramolecular polymers. *Chem. Comm.* **2021***, 57*, 4500–4503.

(S4) Gupta, R. K.; Pathak, S. K.; Pradhan, B.; Rao, D. S. S.; Prasad, S. K.; Achalkumar, A. S. Self-assembly of luminescent N-annulated perylene tetraesters into fluid columnar phases. *Soft Matter* **2015**, *11*, 3629–3636; b) Gupta, R. K.; Rao, D. D. S.; Prasad, S. K.; Achalkumar, A. S. Columnar Self-Assembly of Electron-Deficient Dendronized Bay-Annulated Perylene Bisimides. *Chem. Eur. J.* **2018**, *24*, 3566–3575.

(S5) (a) Pracht, P.; Bohle, F.; Grimme, S. Automated exploration of the low-energy chemical space with fast quantum chemical methods. *Phys. Chem. Chem. Phys.* **2020**, *22*, 7169–7192. (b) Bannwarth, C.; Caldeweyher, E.; Ehlert, S.; Hansen, A.; Pracht, P.; Seibert, J.; Spicher, S.; Grimme, S. Extended tight-binding quantum chemistry methods. *WIREs Comput Mol Sci.* **2021**; 11:e1493.

(S6) Bannwarth, C.; Ehlert, S.; Grimme, S. GFN2-xTB—An Accurate and Broadly Parametrized Self-Consistent Tight-Binding Quantum Chemical Method with Multipole Electrostatics and Density-Dependent Dispersion Contributions. *J. Chem. Theory Comput.* **2019**, *15*, 1652–1671.

(S7) (a) Yang, Y.; Yu, H.; York, D.; Cui, Q.; Elstner, M. Extension of the Self-Consistent-Charge Density-Functional Tight-Binding Method: Third-Order Expansion of the Density Functional Theory Total Energy and Introduction of a Modified Effective Coulomb Interaction. *J. Phys. Chem. A.* **2007**, *111*, 10861-10873. (b) Gaus, M.; Goez, A.; Elstner, M. Parametrization and Benchmark of DFTB3 for Organic Molecules. *J. Chem. Theory Comput.* **2013**, *9*, 338–354.

(S8) Hestand, N. J.; Spano, F. C. Expanded Theory of H- and J-Molecular Aggregates: The Effects of Vibronic Cou-pling and Intermolecular Charge Transfer. *Chem. Rev.* **2018**, *118*, 7069–7163.

(S9) May, V.; Kühn, O. Charge and Energy Transfer Dynamics in Molecular Systems; Wiley-VCH Verlag GmbH & Co. KGaA: Weinheim, Germany, **2011**.

(S10) Gaussian 16, Revision A.03, Frisch, M. J.; Trucks, G. W.; Schlegel, H. B.; Scuseria, G. E.; Robb, M. A.; Cheeseman, J. R.; Scalmani, G.; Barone, V.; Petersson, G. A.; Nakatsuji, H.; Li, X.; Caricato, M.; Marenich, A. V.; Bloino, J.; Janesko, B. G.; Gomperts, R.; Mennucci, B.; Hratchian, H. P.; Ortiz, J. V.; Izmaylov, A. F.; Sonnenberg, J. L.; Williams-Young, D.; Ding, F.; Lipparini, F.; Egidi, F.; Goings, J.; Peng, B.; Petrone, A.; Henderson, T.; Ranasinghe, D.; Zakrzewski, V. G.; Gao, J.; Rega, N.; Zheng, G.; Liang, W.; Hada, M.; Ehara, M.; Toyota, K.; Fukuda, R.; Hasegawa, J.; Ishida, M.; Nakajima, T.; Honda, Y.; Kitao, O.; Nakai, H.; Vreven, T.; Throssell, K.; Montgomery, J. A., Jr.; Peralta, J. E.; Ogliaro, F.; Bearpark, M. J.; Heyd, J. J.; Brothers, E. N.; Kudin, K. N.; Staroverov, V. N.; Keith, T. A.; Kobayashi, R.; Normand, J.; Raghavachari, K.; Rendell, A. P.; Burant, J. C.; Iyengar, S. S.; Tomasi, J.; Cossi, M.; Millam, J. M.; Klene, M.; Adamo, C.; Cammi, R.; Ochterski, J. W.; Martin, R. L.; Morokuma, K.; Farkas, O.; Foresman, J. B.; Fox, D. J. Gaussian, Inc., Wallingford CT, **2016**.

(S11) (a) Lee, C.; Yang, W.; Parr, R. G. Development of the Colle-Salvetti correlation-energy formula into a functional of the electron density. *Phys. Rev. B* **1988**, *37*, 785–789; (b) Becke, A.

D. Density-functional thermochemistry. III. The role of exact exchange. *J. Chem. Phys.* **1993**, *98*, 5648–5652.

(S12) (a) Chai, D.; Head-Gordon, M. Long-range corrected hybrid density functionals with damped atom–atom dispersion corrections. *Phys. Chem. Chem. Phys.* **2008**, *10*, 6615-6620. (b) Stein, T.; Kronik, L.; Baer, R. Reliable Prediction of Charge Transfer Excitations in Molecular Complexes Using Time-Dependent Density Functional Theory. *J. Am. Chem. Soc.* **2009**, *131*, 2818–2820.

(S13) Francl, M. M.; Pietro, W. J.; Hehre, W. J.; Binkley, J. S.; Gordon, M. S.; Defrees, D. J.; Pople, J. A. Self-consistent molecular orbital methods. XXIII. A polarization-type basis set for second-row elements. *J. Chem. Phys.* **1982**, *77*, 3654–3665.

(S14) (a) Tomasi, J.; Persico, M. Molecular Interactions in Solution: An Overview of Methods Based on Continuous Distributions of the Solvent. *Chem. Rev.* **1994**, *94*, 2027–2094. (b) Tomasi, J.; Mennucci, B.; Cammi, R. Quantum Mechanical Continuum Solvation Models. *Chem. Rev.* **2005**, *105*, 2999–3093.

(S15) Canola, S.; Bagnara, G.; Dai, Y.; Ricci, G.; Calzolari, A.; Negri, F. Addressing the Frenkel and charge transfer character of exciton states with a model Hamiltonian based on dimer calculations: Application to large aggregates of perylene bisimide. *J. Chem. Phys.* **2021**, *154*, 124101.

(S16) Curutchet, C.; Mennucci, B. Toward a Molecular Scale Interpretation of Excitation Energy Transfer in Solvated Bichromophoric Systems. *J. Am. Chem. Soc.* **2005**, *127*, 16733–16744.

(S17) Baumeier, B.; Kirkpatrick, J.; Andrienko, D. Density-functional based determination of intermolecular charge transfer properties for large-scale morphologies. *Phys. Chem. Chem. Phys.* **2010**, *12*, 11103–11113.
